# Supplementary material for: Systematic characterization of chromodomain proteins reveals an H3K9me1/2 reader regulating aging in C. elegans
Source: Nat Commun. 2023 Mar 6;14:1254. doi: 10.1038/s41467-023-36898-y (PMC9988841; doi:10.1038/s41467-023-36898-y)
Supplement: Supplementary file 3 — Description of Additional Supplementary Files [file 41467_2023_36898_MOESM3_ESM.pdf]

Description of Additional Supplementary Files

File Name: Supplementary Data 1
Description: ChIP-seq datasets used in this work.

File Name: Supplementary Data 2
Description: Summary of chromodomain protein ChIP-seq peaks called by MACS2.

File Name: Supplementary Data 3
Description: Information of the epigenetic regulatory network.

File Name: Supplementary Data 4
Description: List of genes used in the candidate-based RNAi screening.
