# Supplementary material for: Systematic characterization of chromodomain proteins reveals an H3K9me1/2 reader regulating aging in C. elegans
Source: Nat Commun. 2023 Mar 6;14:1254. doi: 10.1038/s41467-023-36898-y (PMC9988841; doi:10.1038/s41467-023-36898-y)
Supplement: Supplementary file 8 — Reporting Summary [file 41467_2023_36898_MOESM8_ESM.pdf]

nature portfolio

Corresponding author(s): Professor Shouhong Guang

Last updated by author(s): Jan 28, 2023

Reporting Summary

Nature Portfolio wishes to improve the reproducibility of the work that we publish. This form provides structure for consistency and transparency in reporting. For further information on Nature Portfolio policies, see our Editorial Policies and the Editorial Policy Checklist.

Statistics

For all statistical analyses, confirm that the following items are present in the figure legend, table legend, main text, or Methods section.

n/a	Confirmed	
☐	☒	The exact sample size (n) for each experimental group/condition, given as a discrete number and unit of measurement
☐	☒	A statement on whether measurements were taken from distinct samples or whether the same sample was measured repeatedly
☐	☒	The statistical test(s) used AND whether they are one- or two-sided<br>*Only common tests should be described solely by name; describe more complex techniques in the Methods section.*
☒	☐	A description of all covariates tested
☐	☒	A description of any assumptions or corrections, such as tests of normality and adjustment for multiple comparisons
☐	☒	A full description of the statistical parameters including central tendency (e.g. means) or other basic estimates (e.g. regression coefficient) AND variation (e.g. standard deviation) or associated estimates of uncertainty (e.g. confidence intervals)
☐	☒	For null hypothesis testing, the test statistic (e.g. F, t, r) with confidence intervals, effect sizes, degrees of freedom and P value noted<br>*Give P values as exact values whenever suitable.*
☒	☐	For Bayesian analysis, information on the choice of priors and Markov chain Monte Carlo settings
☒	☐	For hierarchical and complex designs, identification of the appropriate level for tests and full reporting of outcomes
☐	☒	Estimates of effect sizes (e.g. Cohen's d, Pearson's r), indicating how they were calculated

Our web collection on statistics for biologists contains articles on many of the points above.

Software and code

Policy information about availability of computer code

Data collection	Leica LAS Microscope Software (version X) were used to acquire images. ChIP-seq was performed on HiSeq1500 system.
Data analysis	Quantification of GFP intensities was performed by ImageJ (v1.8.0). ChIP-seq analysis used Bowtie2 version 2.3.5.1, SAMtools version 0.1.19, MACS2 version 2.1.1, bamCoverage (version 3.5.0), IntervalStats(without a version number), Gephi version 0.9.2

For manuscripts utilizing custom algorithms or software that are central to the research but not yet described in published literature, software must be made available to editors and reviewers. We strongly encourage code deposition in a community repository (e.g. GitHub). See the Nature Portfolio guidelines for submitting code & software for further information.

Data

Policy information about availability of data

All manuscripts must include a data availability statement. This statement should provide the following information, where applicable:

- Accession codes, unique identifiers, or web links for publicly available datasets
- A description of any restrictions on data availability
- For clinical datasets or third party data, please ensure that the statement adheres to our policy

All the high throughput data were deposited onto the Genome Sequence Archive in the National Genomics Data Center, China National Center for Bioinformation/ Beijing Institute of Genomics, Chinese Academy of Sciences (GSA: CRA009179), which are publicly accessible at https://bigd.big.ac.cn/gsa/browse/CRA009179. Downloaded data are from NCBI GEO or modENCODE databases. Links are as below: https://sra-downloadb.be-md.ncbi.nlm.nih.gov/sos1/sra-pub-run-5/

SRR1198654/SRR1198654.1
https://sra-downloadb.be-md.ncbi.nlm.nih.gov/sos1/sra-pub-run-5/SRR1198656/SRR1198656.1
https://sra-downloadb.be-md.ncbi.nlm.nih.gov/sos1/sra-pub-run-5/SRR1198412/SRR1198412.1
https://sra-downloadb.be-md.ncbi.nlm.nih.gov/sos2/sra-pub-run-9/SRR5272577/SRR5272577.1
https://sra-downloadb.be-md.ncbi.nlm.nih.gov/sos3/sra-pub-run-21/SRR10533867/SRR10533867.1
https://sra-downloadb.be-md.ncbi.nlm.nih.gov/sos2/sra-pub-run-9/SRR5272583/SRR5272583.1
Rep-1:ChIP:Cele_WS220:modENCODE_3573:SRR190688.fast.fastq.gz
https://sra-downloadb.be-md.ncbi.nlm.nih.gov/sos1/sra-pub-run-5/SRR1198473/SRR1198473.1
Rep-1:ChIP:Cele_WS220:modENCODE_5152:084_H3K18ac_Eemb131A_M11.fastq.gz
Rep-1:ChIP:Cele_WS220:modENCODE_5159:13_WA30634849_H3K27ac_8002_Eemb14A_M4.fastq.gz
Rep-1:ChIP:Cele_WS220:modENCODE_5160:14_UP07448_H3K27me1_24439_Eemb14A_M5.fastq.gz
Rep-1:ChIP:Cele_WS220:modENCODE_5044:12-5_ACAGTG_L008.fastq.gz
https://sra-downloadb.be-md.ncbi.nlm.nih.gov/sos2/sra-pub-run-13/SRR7164188/SRR7164188.1
https://sra-downloadb.be-md.ncbi.nlm.nih.gov/sos2/sra-pub-run-13/SRR7164198/SRR7164198.1
ChIP-seq:Rep-1:ChIP:Cele_WS220:modENCODE_5043:H3K36ac_novusNB211254_FE1_ACAGTG_L004.fastq.gz
Rep-1:ChIP:Cele_WS220:modENCODE_6244:9_seq-Ab9048_H3K36me1_fem-2[b245]_AD_2_csr2.fastq.gz
Rep-1:ChIP:Cele_WS220:modENCODE_5164:18_HK00012_H3K36me2_2C3_Eemb14A_M7.fastq.gz
Rep-1:ChIP:Cele_WS220:modENCODE_5041:H3K36me2_Kimura2C3_ME5_CGATGT_L002.fastq.gz
https://sra-downloadb.be-md.ncbi.nlm.nih.gov/sos2/sra-pub-run-13/SRR7164176/SRR7164176.1
https://sra-downloadb.be-md.ncbi.nlm.nih.gov/sos2/sra-pub-run-13/SRR7164186/SRR7164186.1
https://sra-downloadb.be-md.ncbi.nlm.nih.gov/sos2/sra-pub-run-13/SRR7164164/SRR7164164.1
Rep-1:ChIP:Cele_WS220:modENCODE_5157:11_WA30834809_H3K4me2_8002_Eemb13A_M2.fastq.gz
Rep-1:ChIP:Cele_WS220:modENCODE_5055:ME5_CGATGT_L003.fastq.gz
https://sra-downloadb.be-md.ncbi.nlm.nih.gov/sos2/sra-pub-run-13/SRR7164152/SRR7164152.1
https://sra-downloadb.be-md.ncbi.nlm.nih.gov/sos2/sra-pub-run-13/SRR7164162/SRR7164162.1
Rep-1:ChIP:Cele_WS220:modENCODE_5169:7_ab3594_H3K79me2_346021_Eemb132A_M13.fastq.gz
https://sra-downloadb.be-md.ncbi.nlm.nih.gov/sos1/sra-pub-run-5/SRR1030715/SRR1030715.1
Rep-1:ChIP:Cele_WS220:modENCODE_3578:IL2_F_N2_L3_raw_NA_NA_IL015_Rab03283.fastq.gz
Rep-1:ChIP:Cele_WS220:modENCODE_5149:079_H3K9acS10ph_03894_NoIndex_L001_R1_001_M2.fastq.gz
Rep-1:ChIP:Cele_WS220:modENCODE_5988:4_F_N2_L3_raw_NA_NA_AA134_Fxx02819.fastq.gz
Rep-1:ChIP:Cele_WS220:modENCODE_5171:9_ab8896_H3K9me1_104560_Eemb132A_M7.fastq.gz
Rep-1:ChIP:Cele_WS220:modENCODE_5036:090512_AA1_2_CGATGT_L001.fastq.gz
https://sra-downloadb.be-md.ncbi.nlm.nih.gov/sos3/sra-pub-run-21/SRR8602973/SRR8602973.1
https://sra-downloadb.be-md.ncbi.nlm.nih.gov/sos2/sra-pub-run-9/SRR4319281/SRR4319281.1
https://sra-downloadb.be-md.ncbi.nlm.nih.gov/sos1/sra-pub-run-1/SRR8201366/SRR8201366.1
https://sra-downloadb.be-md.ncbi.nlm.nih.gov/sos2/sra-pub-run-9/SRR5297105/SRR5297105.1
Rep-1:ChIP:Cele_WS220:modENCODE_5244:WA30335199_H3S10ph_csr3.fastq.gz
Rep-1:ChIP:Cele_WS220:modENCODE_3572:seq-LPAR109_H4tetraac_109_N2_L3_2.f.fastq.gz
https://sra-downloadb.be-md.ncbi.nlm.nih.gov/sos2/sra-pub-run-11/SRR5758242/SRR5758242.1
https://sra-downloadb.be-md.ncbi.nlm.nih.gov/sos1/sra-pub-run-5/SRR947588/SRR947588.1
https://sra-downloadb.be-md.ncbi.nlm.nih.gov/sos3/sra-pub-run-21/SRR10533859/SRR10533859.1
https://sra-downloadb.be-md.ncbi.nlm.nih.gov/sos2/sra-pub-run-13/SRR7191148/SRR7191148.1
https://sra-downloadb.be-md.ncbi.nlm.nih.gov/sos2/sra-pub-run-9/SRR4319285/SRR4319285.1
Rep-1:ChIP:Cele_WS220:modENCODE_5976:08_E_N2_L3_raw_NA_NA_PK011_Fxx00617.fastq.gz
https://sra-downloadb.be-md.ncbi.nlm.nih.gov/sos1/sra-pub-run-2/SRR800682/SRR800682.2
https://sra-downloadb.be-md.ncbi.nlm.nih.gov/sos1/sra-pub-run-1/SRR8176688/SRR8176688.1
https://sra-downloadb.be-md.ncbi.nlm.nih.gov/sos2/sra-pub-run-9/SRR4319287/SRR4319287.1
https://sra-downloadb.be-md.ncbi.nlm.nih.gov/sos2/sra-pub-run-9/SRR4319289/SRR4319289.1
https://sra-downloadb.be-md.ncbi.nlm.nih.gov/sos2/sra-pub-run-9/SRR5272589/SRR5272589.1
https://sra-downloadb.be-md.ncbi.nlm.nih.gov/sos2/sra-pub-run-9/SRR5272609/SRR5272609.1
https://sra-downloadb.be-md.ncbi.nlm.nih.gov/sos2/sra-pub-run-9/SRR4319291/SRR4319291.1
https://sra-downloadb.be-md.ncbi.nlm.nih.gov/sos2/sra-pub-run-9/SRR5272593/SRR5272593.1
https://www.encodeproject.org/files/ENCFF791ASR/download/ENCFF791ASR.fastq.gz
https://sra-downloadb.be-md.ncbi.nlm.nih.gov/sos1/sra-pub-run-1/SRR107554/SRR107554.2
https://sra-downloadb.be-md.ncbi.nlm.nih.gov/sos2/sra-pub-run-9/SRR4319293/SRR4319293.1
https://sra-downloadb.be-md.ncbi.nlm.nih.gov/sos2/sra-pub-run-13/SRR6760670/SRR6760670.1
Rep-1:ChIP:Cele_WS220:modENCODE_5985:3_E_N2_L3_raw_NA_NA_AA010_Fxx00357.fastq.gz
https://www.encodeproject.org/files/ENCFF020ZSR/download/ENCFF020ZSR.fastq.gz
https://sra-downloadb.be-md.ncbi.nlm.nih.gov/sos2/sra-pub-run-11/SRR5766320/SRR5766320.1
https://sra-downloadb.be-md.ncbi.nlm.nih.gov/sos2/sra-pub-run-11/SRR5766322/SRR5766322.1
https://sra-downloadb.be-md.ncbi.nlm.nih.gov/sos2/sra-pub-run-13/SRR7191144/SRR7191144.1

Human research participants

Policy information about studies involving human research participants and Sex and Gender in Research.

Reporting on sex and gender: No human research participants were involved.

Population characteristics: No human research participants were involved.

Recruitment: No human research participants were involved.

Ethics oversight	No human research participants were involved.

Note that full information on the approval of the study protocol must also be provided in the manuscript.

Field-specific reporting

Please select the one below that is the best fit for your research. If you are not sure, read the appropriate sections before making your selection.

☒ Life sciences ☐ Behavioural & social sciences ☐ Ecological, evolutionary & environmental sciences

For a reference copy of the document with all sections, see nature.com/documents/nr-reporting-summary-flat.pdf

Life sciences study design

All studies must disclose on these points even when the disclosure is negative.

Sample size	For experiments involving imaging and quantification, numbers of animals were > 50.
Data exclusions	No data were excluded from the analysis.
Replication	Gene expression evaluation and phenotype experiments were repeated at least 3 times. All attempts of replication were successful.
Randomization	Animals used in the experiments were cultured simultaneously on the same plates and selected randomly to control or experimental groups.
Blinding	The investigators were blinded to group allocation during data collection and analysis.

Reporting for specific materials, systems and methods

We require information from authors about some types of materials, experimental systems and methods used in many studies. Here, indicate whether each material, system or method listed is relevant to your study. If you are not sure if a list item applies to your research, read the appropriate section before selecting a response.

Materials & experimental systems

n/a	Involved in the study	
☐	☒	Antibodies
☒	☐	Eukaryotic cell lines
☒	☐	Palaeontology and archaeology
☐	☒	Animals and other organisms
☒	☐	Clinical data
☒	☐	Dual use research of concern

Methods

n/a	Involved in the study	
☐	☒	ChIP-seq
☒	☐	Flow cytometry
☒	☐	MRI-based neuroimaging

Antibodies

Antibodies used	Anti-GFP antibody (abcam ab290, lot # GR3320917-1), used at 1.5:1000
Validation	Anti-GFP antibody (abcam ab290) antibody has been validated by abcam by demonstrating Western blot and Immunocytochemistry (see website). This antibody was also validated in our previous works (Weng et al., 2019 and Huang et al., 2021).

Animals and other research organisms

Policy information about studies involving animals; ARRIVE guidelines recommended for reporting animal research, and Sex and Gender in Research

Laboratory animals	Caenorhabditis elegans. Adult (3 day aged) and late embryo (within 9 hours before hatched) staged animals were used in ChIP-seq.
Wild animals	No wild animals were used in this study.
Reporting on sex	Hermaphrodite animals were used in this study.
Field-collected samples	No field-collected samples were used in this study.

Ethics oversight	Caenorhabditis elegans was used in this study and no ethical approval was required.

Note that full information on the approval of the study protocol must also be provided in the manuscript.

ChIP-seq

Data deposition

- [x] Confirm that both raw and final processed data have been deposited in a public database such as GEO.
- [ ] Confirm that you have deposited or provided access to graph files (e.g. BED files) for the called peaks.

Data access links<br>*May remain private before publication.*	All the high throughput data were deposited onto the Genome Sequence Archive in the National Genomics Data Center, China National Center for Bioinformation/Beijing Institute of Genomics, Chinese Academy of Sciences (GSA: CRA009179), which are publicly accessible at https://bigd.big.ac.cn/gsa/browse/CRA009179.
Files in database submission	CHD1_ChIPseq<br>CHD1_input<br>CHD3_ChIPseq<br>CHD3_input<br>CHD7_ChIPseq<br>CHD7_input<br>LET418_ChIPseq<br>LET418_input<br>CEC2_ChIPseq<br>CEC2_input<br>CEC5_ChIPseq<br>CEC5_input<br>CEC7_ChIPseq<br>CEC7_input<br>CEC8_ChIPseq<br>CEC8_input<br>CEC10_ChIPseq<br>CEC10_input<br>SET31_ChIPseq<br>SET31_input<br>UAD2_ChIPseq<br>UAD2_input<br>CEC5_WT_ChIPseq<br>CEC5_WT_input<br>CEC5_set25_ChIPseq<br>CEC5_set25_input<br>CEC5_met2_ChIPseq<br>CEC5_met2_input<br>CEC5_met2_set25_ChIPseq<br>CEC5_met2_set25_input<br>CEC5_ust283_ChIPseq<br>CEC5_ust283_input
Genome browser session<br>(e.g. UCSC)	no longer applicable

Methodology

Replicates	CEC-5 GFP(A) has two replicates. ChIP-seq for other chromodomain factors was performed once.
Sequencing depth	10M raw reads, single-end, 50 bp length
Antibodies	Anti-GFP antibody (abcam ab290, lot # GR3320917-1), used at 1.5:1000
Peak calling parameters	ChIP-seq peaks were called using MACS2 version 2.1.1 (ZHANG et al. 2008) with subcommand callpeak with defined parameters (-g ce -B -f BAM -q 0.01 -m 4 50).
Data quality	Quality of each chromodomain protein ChIP-seq dataset used in this work was assessed. Firstly, the ratio of reads falling within peak regions of each sample was calculated and defined as Fraction of Reads in Peaks (FRiP) (LANDT et al. 2012). Secondly, cross-correlation analysis was performed (KHARCHENKO et al. 2008; LANDT et al. 2012). Thirdly, MA-plots were included to show the enrichment of each chromodomain protein versus input.
Software	Bowtie2 version 2.3.5.1; SAMtools version 0.1.19; MACS2 version 2.1.1; Deeptools subcommand bamCoverage (version 3.5.0); R package ChIPseeker (Yu et al., 2015); IntervalStats; Gephi.
