# Supplementary material for: Systematic characterization of chromodomain proteins reveals an H3K9me1/2 reader regulating aging in C. elegans
Source: Nat Commun. 2023 Mar 6;14:1254. doi: 10.1038/s41467-023-36898-y (PMC9988841; doi:10.1038/s41467-023-36898-y)
Supplement: Supplementary file 1 — Supplementary Information [file 41467_2023_36898_MOESM1_ESM.pdf]

Supplementary Information

Systematic characterization of chromodomain proteins reveals an H3K9me1/2 reader regulating aging in *C. elegans*

Xinhao Hou[ǂ], Mingjing Xu[ǂ], Chengming Zhu[ǂ], Jianing Gao, Meili Li, Xiangyang Chen, Cheng Sun, Björn Nashan, Jianye Zang, Ying Zhou*, Shouhong Guang*, and Xuezhu Feng*

[ǂ]These authors contributed equally to the work.
*Correspondence should be addressed to caddiezy@ustc.edu.cn, sguang@ustc.edu.cn, and fengxz@ustc.edu.cn.

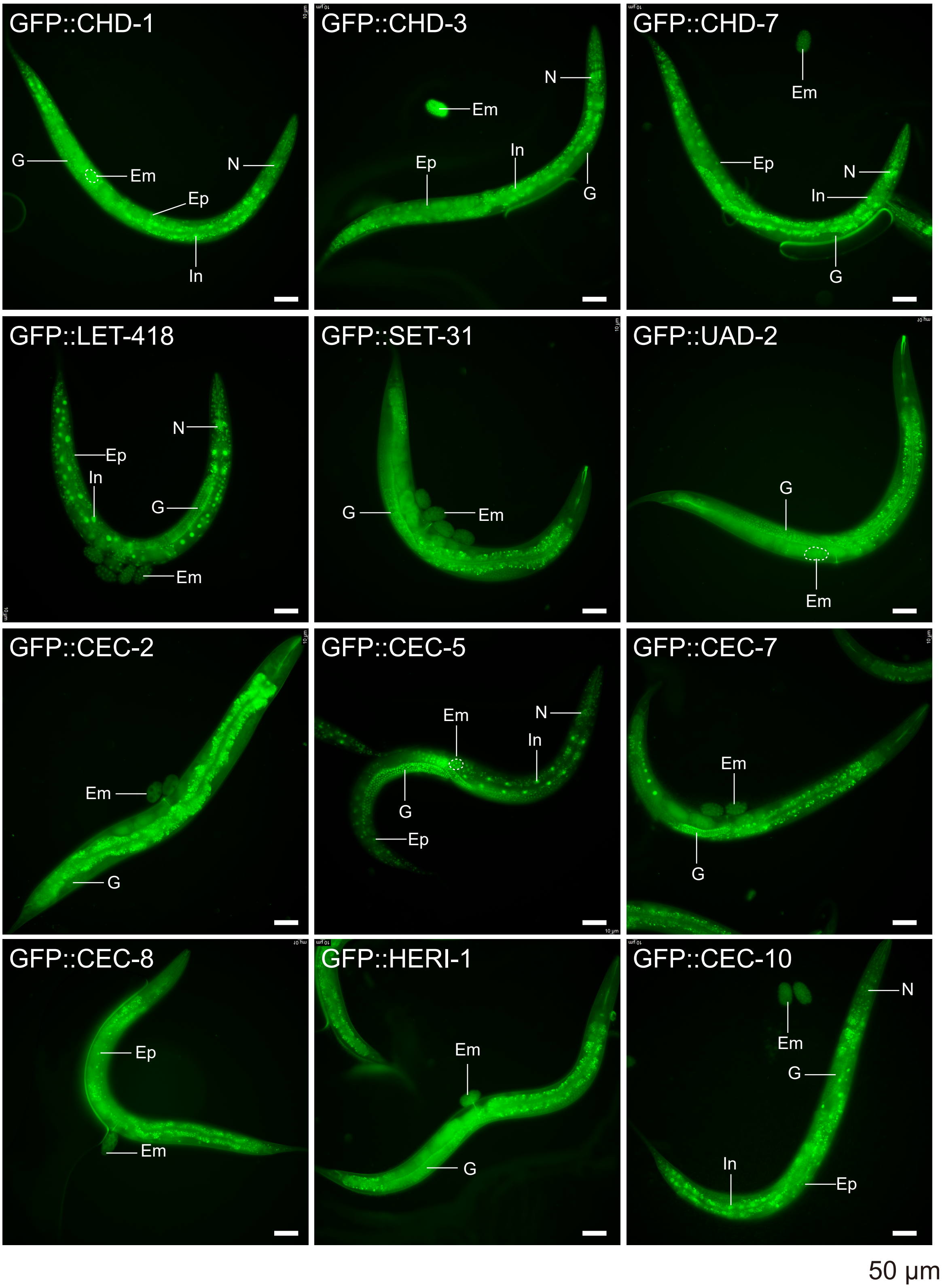

Supplementary Figure 1. Expression patterns of chromodomain proteins throughout the whole body. Overview of the expression of indicated chromodomain proteins throughout the whole body. N, neuron; G, germ line; In, intestine; Em, embryo; Ep, epidermis (scale bar, 50 μm).

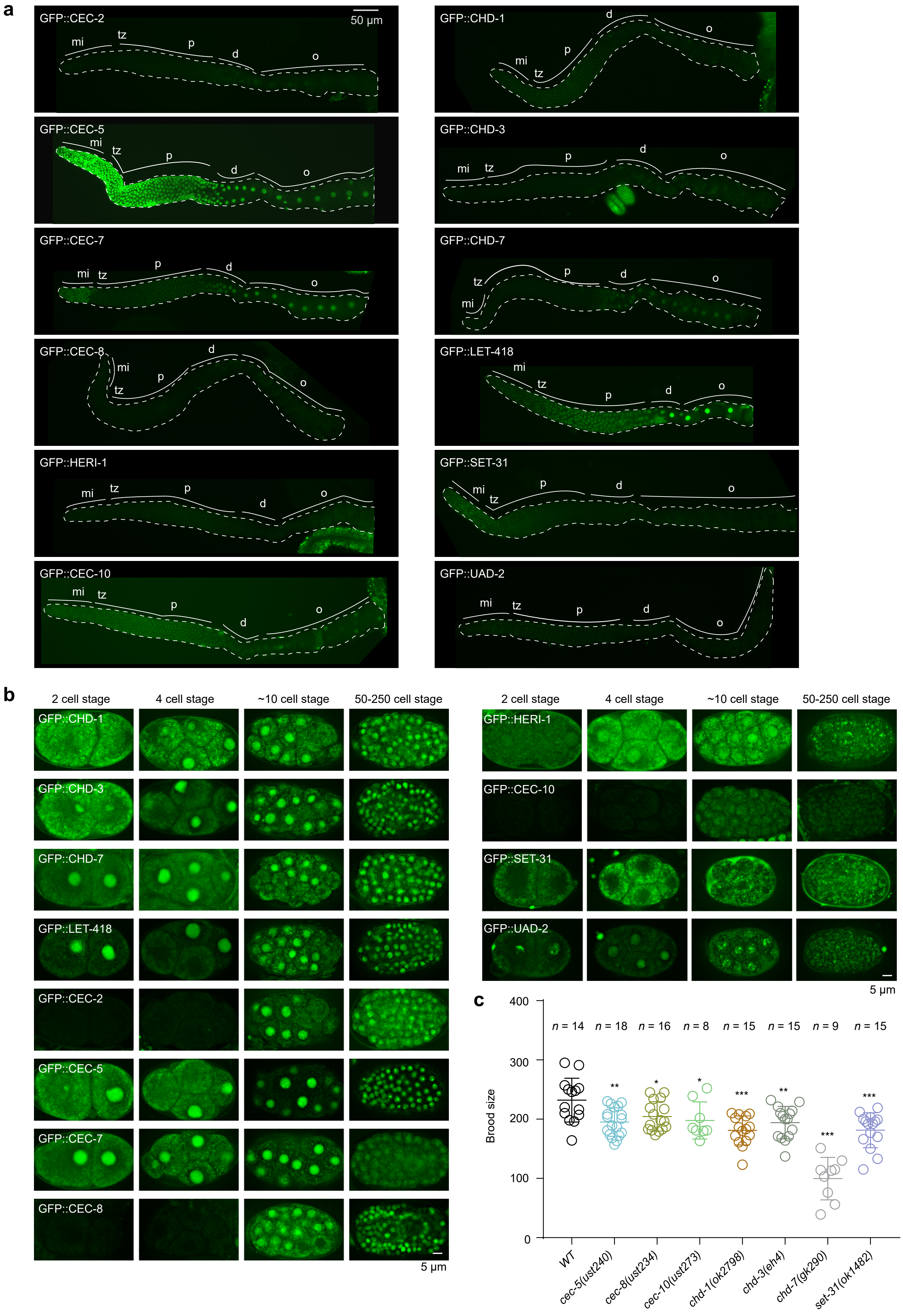
a
GFP::CEC-2
50 μm
GFP::CEC-5
GFP::CEC-7
GFP::CEC-8
GFP::HERI-1
GFP::CEC-10
GFP::CHD-1
GFP::CHD-3
GFP::CHD-7
GFP::LET-418
GFP::SET-31
GFP::UAD-2
mi
tz
p
d
o
b
2 cell stage
4 cell stage
~10 cell stage
50-250 cell stage
5 μm
c
Brood size
n = 14
n = 18
n = 16
n = 8
n = 15
n = 15
n = 9
n = 15
WT
cec-5(ust240)
cec-8(ust234)
cec-10(ust273)
chd-1(ok2798)
chd-3(eh4)
chd-7(gk290)
set-31(ok1482)

Supplementary Figure 2. Expression of chromodomain proteins in germline and embryos. (a-b) Expression patterns of chromodomain proteins in germline **(a)** and embryos **(b)**. The expression of a representative image for each chromodomain protein in the indicated tissue or development stage is shown. Germline is indicated by white dashed line. mi, mitotic region; tz, transition zone; p, pachytene; d, diplotene; o, oocyte. (Germline, scale bar, 50 μm; embryos, scale bar, 5 μm). **(c)** Brood size of the indicated animals at 20°C. n, the number of animals tested. Mean ± SD. Two-sided t-test was performed. ***$p < 0.001$, **$0.001 < p < 0.01$, *$0.01 < p < 0.05$. *cec-5(ust240)*, p=0.0019; *cec-8(ust234)*, p=0.0224; *cec-10(ust273)*, p=0.0399; *chd-1(ok2798)*, p=0.0001; *chd-3(eh4)*, p=0.0039; *chd-7(gk290)*, $p=3.28 \times 10^{-8}$; *set-31(ok1482)*, p=0.0003. Source data are provided as a Source Data file.

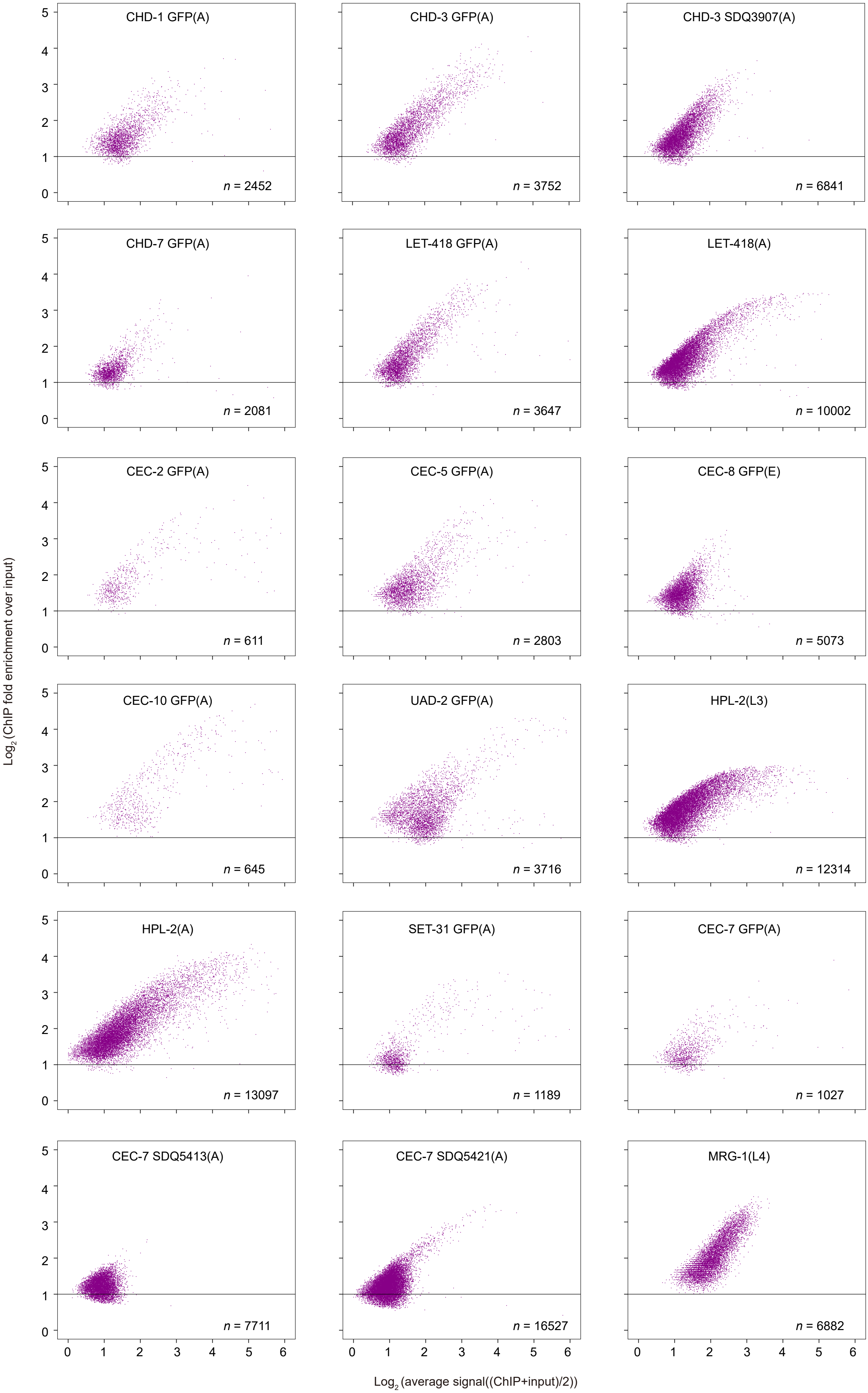
CHD-1 GFP(A)
n = 2452
CHD-3 GFP(A)
n = 3752
CHD-3 SDQ3907(A)
n = 6841
CHD-7 GFP(A)
n = 2081
LET-418 GFP(A)
n = 3647
LET-418(A)
n = 10002
CEC-2 GFP(A)
n = 611
CEC-5 GFP(A)
n = 2803
CEC-8 GFP(E)
n = 5073
CEC-10 GFP(A)
n = 645
UAD-2 GFP(A)
n = 3716
HPL-2(L3)
n = 12314
HPL-2(A)
n = 13097
SET-31 GFP(A)
n = 1189
CEC-7 GFP(A)
n = 1027
CEC-7 SDQ5413(A)
n = 7711
CEC-7 SDQ5421(A)
n = 16527
MRG-1(L4)
n = 6882
Log2 (ChIP fold enrichment over input)
Log2 (average signal((ChIP+input)/2))

Supplementary Figure 3. Quality control of ChIP-seq datasets. MA-plots displaying log_2 enrichment of indicated chromodomain protein versus input. Sites that are identified to be peaks using MACS2 with subcommand callpeak with defined parameters (-g ce -B -f BAM -q 0.01 -m 4 50) are used. Total number of peaks are depicted in each plot.

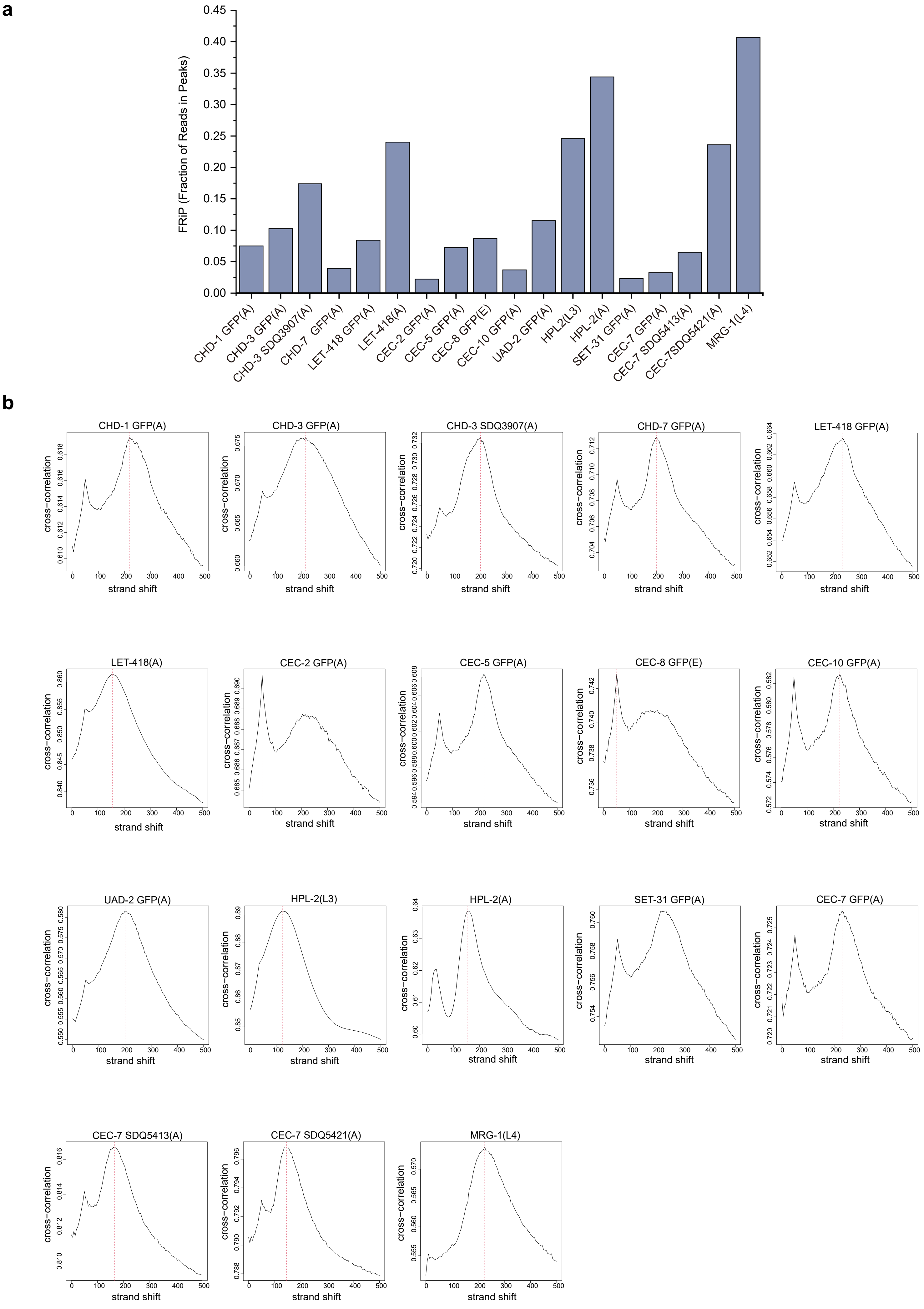
a
FRiP (Fraction of Reads in Peaks)
0.00
0.05
0.10
0.15
0.20
0.25
0.30
0.35
0.40
0.45
CHD-1 GFP(A)
CHD-3 GFP(A)
CHD-3 SDQ3907(A)
CHD-7 GFP(A)
LET-418 GFP(A)
LET-418(A)
CEC-2 GFP(A)
CEC-5 GFP(A)
CEC-8 GFP(E)
CEC-10 GFP(A)
UAD-2 GFP(A)
HPL2(L3)
HPL-2(A)
SET-31 GFP(A)
CEC-7 GFP(A)
CEC-7 SDQ5413(A)
CEC-7SDQ5421(A)
MRG-1(L4)
b
CHD-1 GFP(A)
CHD-3 GFP(A)
CHD-3 SDQ3907(A)
CHD-7 GFP(A)
LET-418 GFP(A)
LET-418(A)
CEC-2 GFP(A)
CEC-5 GFP(A)
CEC-8 GFP(E)
CEC-10 GFP(A)
UAD-2 GFP(A)
HPL-2(L3)
HPL-2(A)
SET-31 GFP(A)
CEC-7 GFP(A)
CEC-7 SDQ5413(A)
CEC-7 SDQ5421(A)
MRG-1(L4)
cross−correlation
strand shift

Supplementary Figure 4. Quality control of ChIP-seq datasets. (a) Fraction of Reads in Peaks (FRiP) of chromodomain proteins. **(b)** Cross-correlation plot files of indicated chromodomain proteins. The normalized ratio between the fragment-length cross-correlation peak and the background cross-correlation (normalized strand coefficient, NSC) and the ratio between the fragment-length peak and the read-length peak (relative strand correlation, RSC) of each chromodomain proteins are listed in Supplementary Table 3.

a

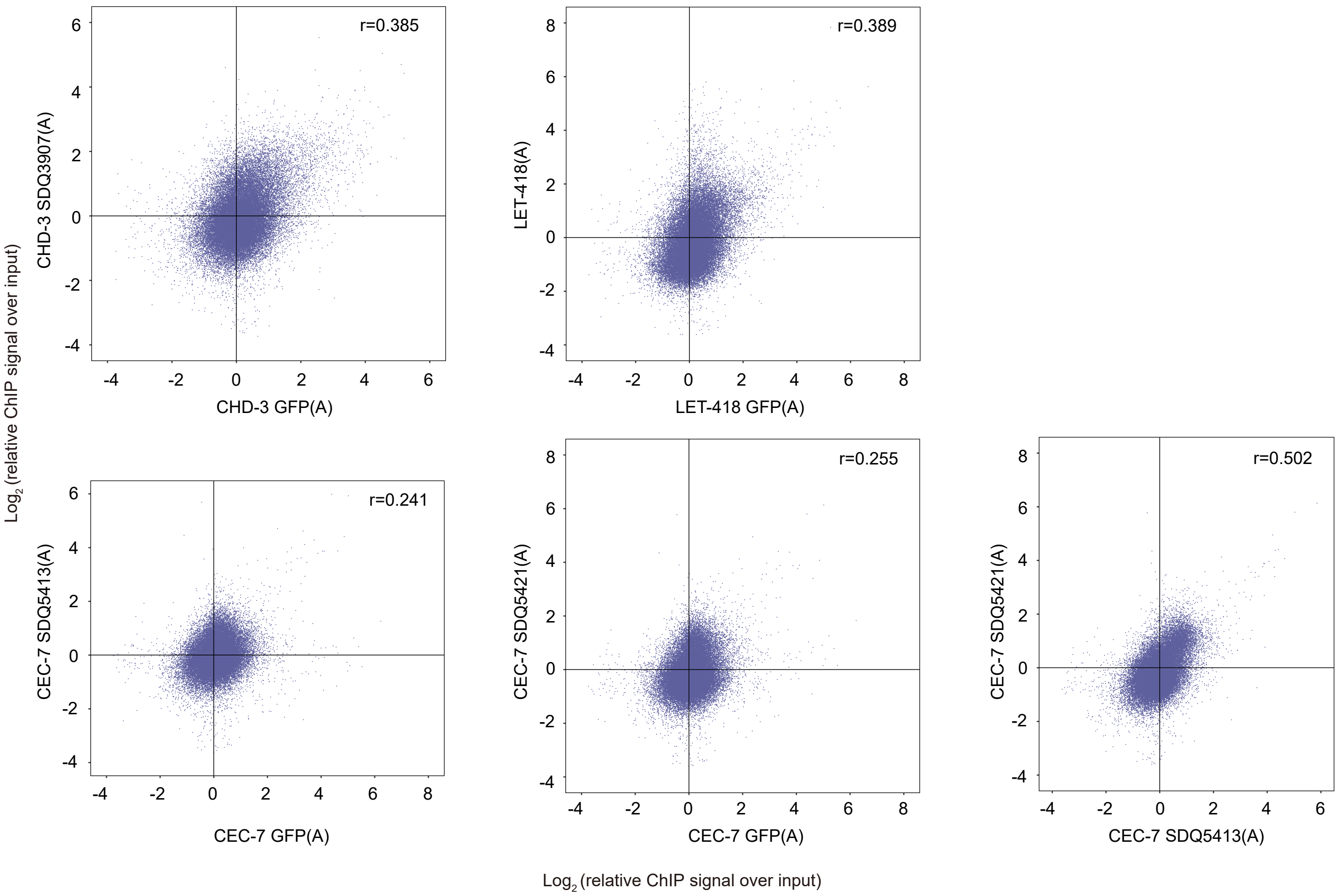
r=0.385
CHD-3 SDQ3907(A)
CHD-3 GFP(A)
r=0.389
LET-418(A)
LET-418 GFP(A)
r=0.241
CEC-7 SDQ5413(A)
CEC-7 GFP(A)
r=0.255
CEC-7 SDQ5421(A)
CEC-7 GFP(A)
r=0.502
CEC-7 SDQ5421(A)
CEC-7 SDQ5413(A)
Log_2(relative ChIP signal over input)
Log_2(relative ChIP signal over input)

b

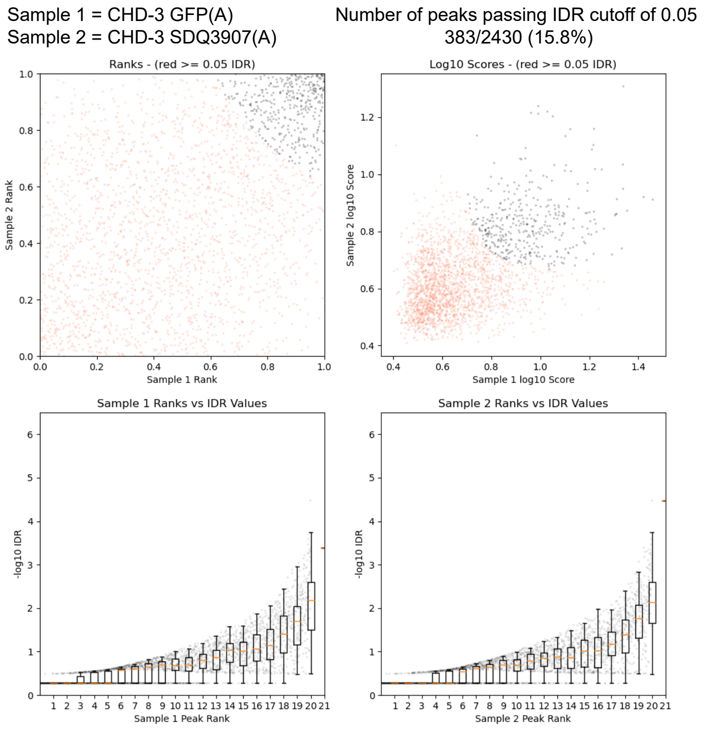
Sample 1 = CHD-3 GFP(A)
Sample 2 = CHD-3 SDQ3907(A)
Number of peaks passing IDR cutoff of 0.05
383/2430 (15.8%)
Ranks - (red >= 0.05 IDR)
Sample 1 Rank
Sample 2 Rank
Log10 Scores - (red >= 0.05 IDR)
Sample 1 log10 Score
Sample 2 log10 Score
Sample 1 Ranks vs IDR Values
Sample 1 Peak Rank
-log10 IDR
Sample 2 Ranks vs IDR Values
Sample 2 Peak Rank
-log10 IDR

c

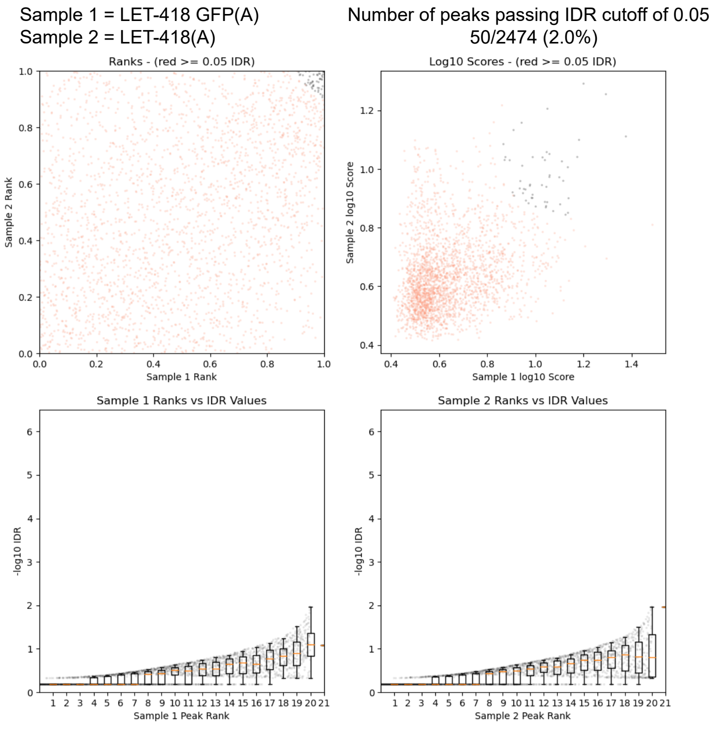
Sample 1 = LET-418 GFP(A)
Sample 2 = LET-418(A)
Number of peaks passing IDR cutoff of 0.05
50/2474 (2.0%)
Ranks - (red >= 0.05 IDR)
Sample 1 Rank
Sample 2 Rank
Log10 Scores - (red >= 0.05 IDR)
Sample 1 log10 Score
Sample 2 log10 Score
Sample 1 Ranks vs IDR Values
Sample 1 Peak Rank
-log10 IDR
Sample 2 Ranks vs IDR Values
Sample 2 Peak Rank
-log10 IDR

d

Sample 1 = CEC-7 GFP(A)
Sample 2 = CEC-7 SDQ5413(A)

Number of peaks passing IDR cutoff of 0.05
85/111 (76.6%)

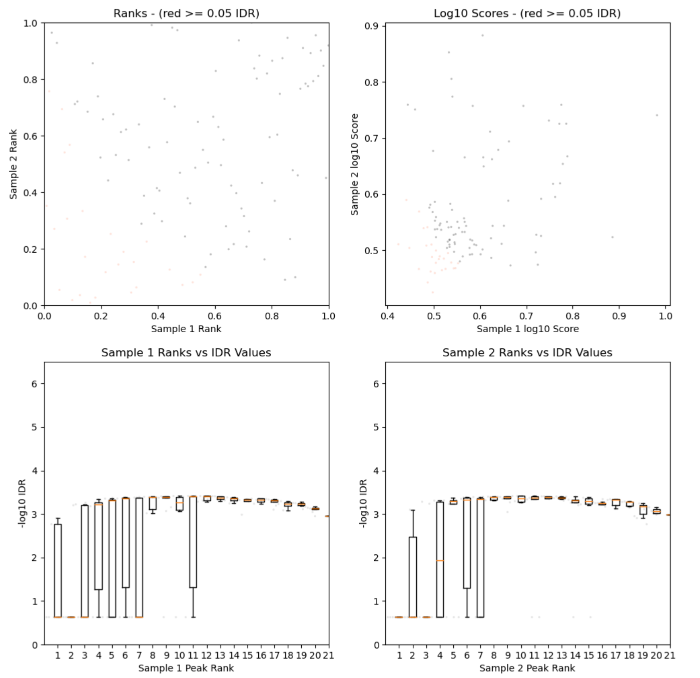

e

Sample 1 = CEC-7 GFP(A)
Sample 2 = CEC-7 SDQ5421(A)

Number of peaks passing IDR cutoff of 0.05
87/477 (18.2%)

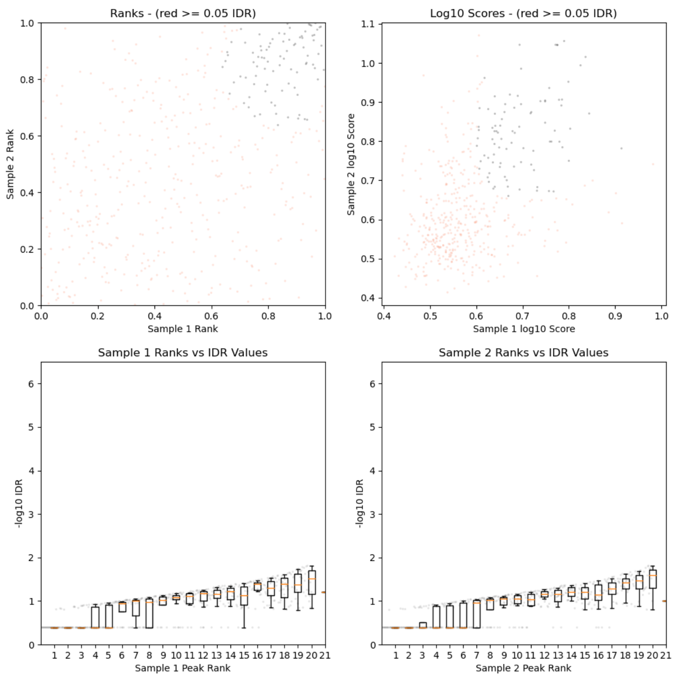

f

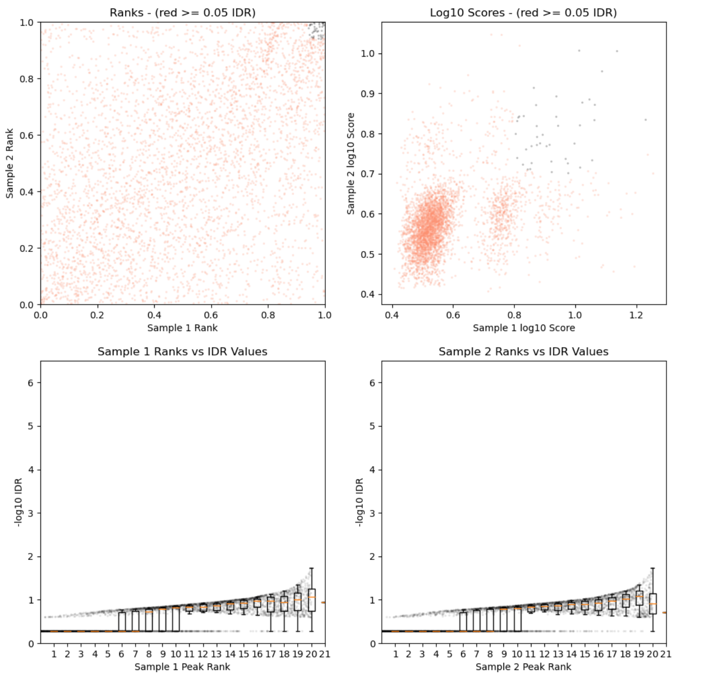

Sample 1 = CEC-7 SDQ5413(A)
Sample 2 = CEC-7 SDQ5421(A)

Number of peaks passing IDR cutoff of 0.05
41/4005 (1.0%)

Supplementary Figure 5. Correlation evaluation of ChIP-seq data in this work and the published datasets. **(a)** Scatter plots comparing $\log_2$ enrichments of indicated pairs of ChIP-seq datasets used in this work. n = 42,496 fragments of the genome. A two-sided t-test was performed. All $p < 2.2 \times 10^{-16}$. r, Pearson correlation coefficient. **(b, c, d, e, f)** The irreproducible discovery rate (IDR) framework for assessing reproducibility of indicated pairs of ChIP-seq datasets used in this work. Number of peaks passing IDR cutoff of 0.05 / number of total peaks used for analysis of each pair is shown. The overlayed boxplots display the distribution of IDR values in each 5% quantile. Maximum and minimum values are shown by whiskers. 75th and 25th percentiles are shown by the bounds of box. 50th percentile is shown by the center of box.

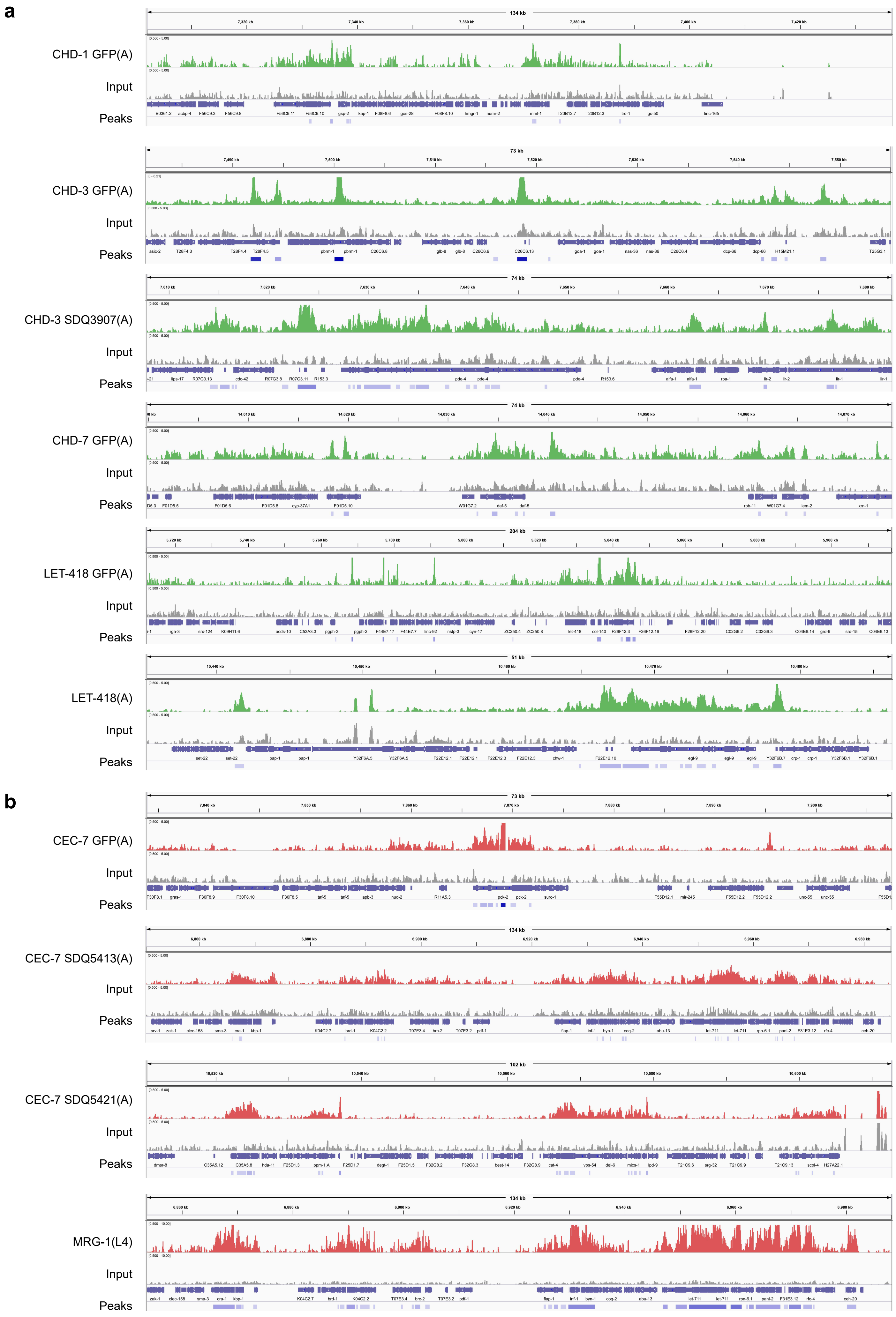
a
CHD-1 GFP(A)
Input
Peaks
CHD-3 GFP(A)
Input
Peaks
CHD-3 SDQ3907(A)
Input
Peaks
CHD-7 GFP(A)
Input
Peaks
LET-418 GFP(A)
Input
Peaks
LET-418(A)
Input
Peaks
b
CEC-7 GFP(A)
Input
Peaks
CEC-7 SDQ5413(A)
Input
Peaks
CEC-7 SDQ5421(A)
Input
Peaks
MRG-1(L4)
Input
Peaks

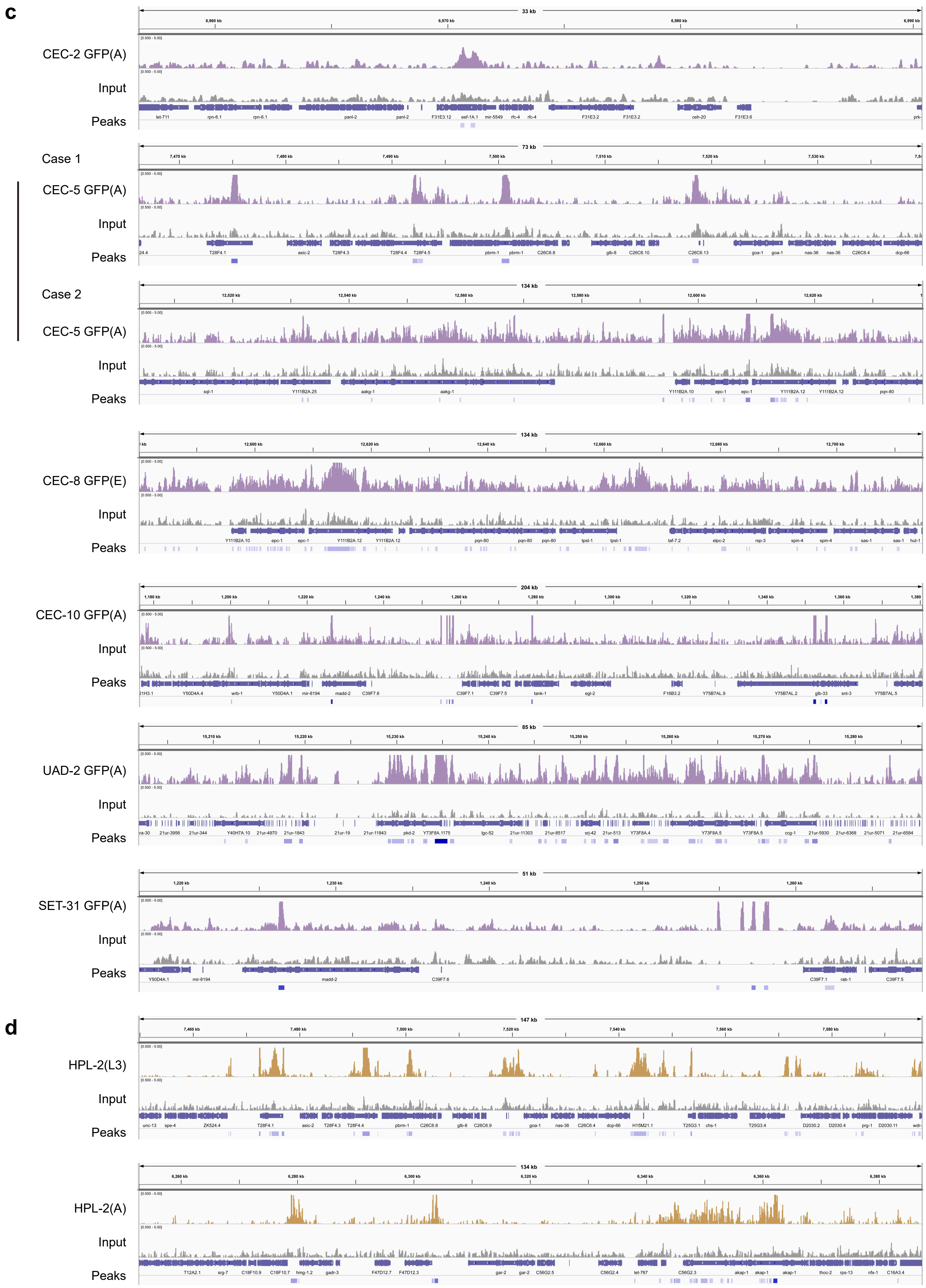

Supplementary Figure 6. Examples of narrow peaks of indicated chromodomain proteins identified by MACS2. (a-d) Representative IGV tracks showing examples of the peaks of chromodomain proteins. CHD family **(a)**, Msl-3 homolog family **(b)**, proteins with no clear homolog in human **(c)**, and HP1 family **(d)**.

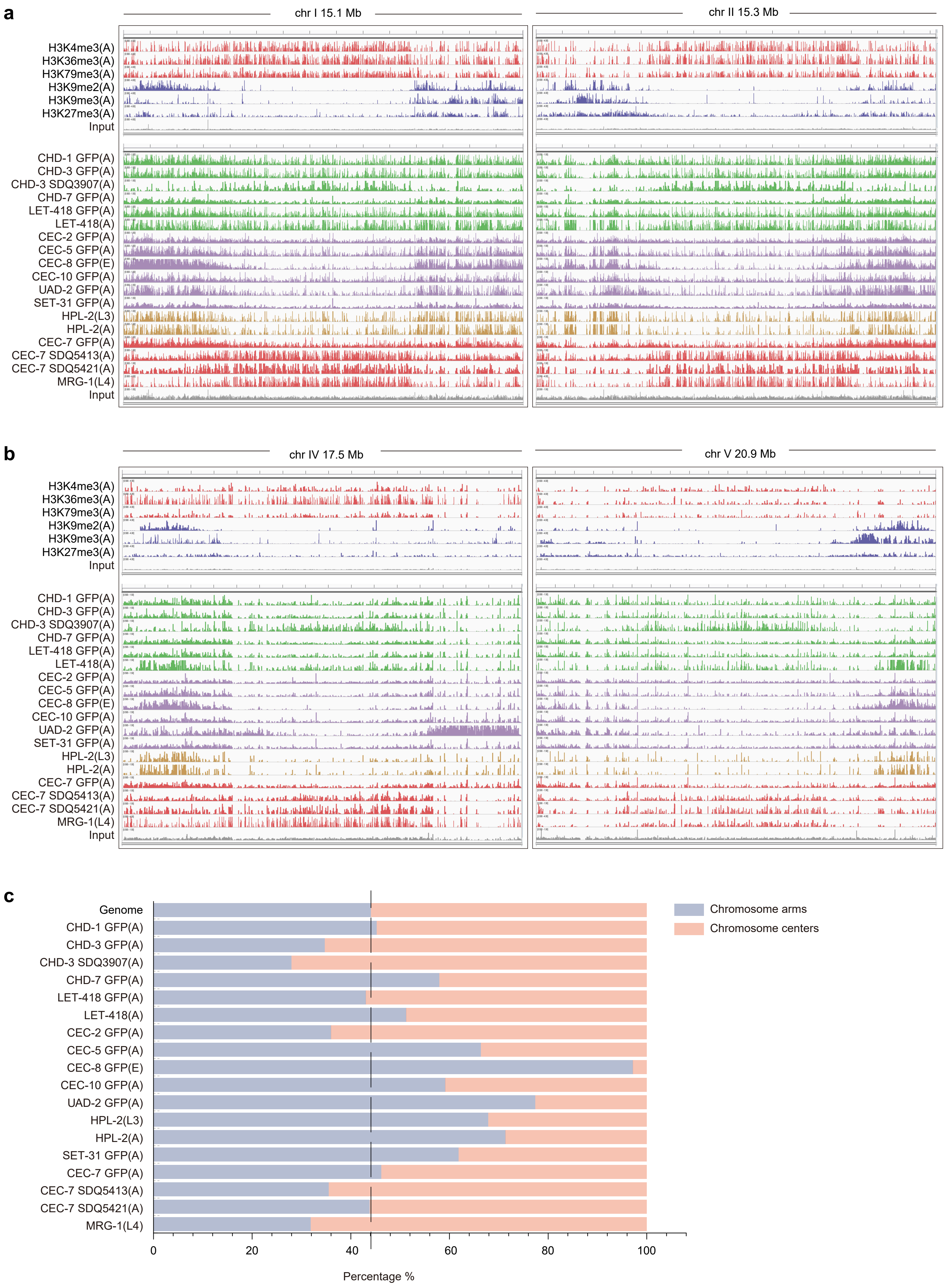
a
chr I 15.1 Mb
chr II 15.3 Mb
H3K4me3(A)
H3K36me3(A)
H3K79me3(A)
H3K9me2(A)
H3K9me3(A)
H3K27me3(A)
Input
CHD-1 GFP(A)
CHD-3 GFP(A)
CHD-3 SDQ3907(A)
CHD-7 GFP(A)
LET-418 GFP(A)
LET-418(A)
CEC-2 GFP(A)
CEC-5 GFP(A)
CEC-8 GFP(E)
CEC-10 GFP(A)
UAD-2 GFP(A)
SET-31 GFP(A)
HPL-2(L3)
HPL-2(A)
CEC-7 GFP(A)
CEC-7 SDQ5413(A)
CEC-7 SDQ5421(A)
MRG-1(L4)
Input
b
chr IV 17.5 Mb
chr V 20.9 Mb
H3K4me3(A)
H3K36me3(A)
H3K79me3(A)
H3K9me2(A)
H3K9me3(A)
H3K27me3(A)
Input
CHD-1 GFP(A)
CHD-3 GFP(A)
CHD-3 SDQ3907(A)
CHD-7 GFP(A)
LET-418 GFP(A)
LET-418(A)
CEC-2 GFP(A)
CEC-5 GFP(A)
CEC-8 GFP(E)
CEC-10 GFP(A)
UAD-2 GFP(A)
SET-31 GFP(A)
HPL-2(L3)
HPL-2(A)
CEC-7 GFP(A)
CEC-7 SDQ5413(A)
CEC-7 SDQ5421(A)
MRG-1(L4)
Input
c
Genome
CHD-1 GFP(A)
CHD-3 GFP(A)
CHD-3 SDQ3907(A)
CHD-7 GFP(A)
LET-418 GFP(A)
LET-418(A)
CEC-2 GFP(A)
CEC-5 GFP(A)
CEC-8 GFP(E)
CEC-10 GFP(A)
UAD-2 GFP(A)
HPL-2(L3)
HPL-2(A)
SET-31 GFP(A)
CEC-7 GFP(A)
CEC-7 SDQ5413(A)
CEC-7 SDQ5421(A)
MRG-1(L4)
Chromosome arms
Chromosome centers
0
20
40
60
80
100
Percentage %

Supplementary Figure 7. Diverse genome distribution of chromodomain proteins in *C. elegans*.
(a-b) Distribution of ChIP-seq signals of active (red), repressive (blue) chromatin marks (upper panel), and chromodomain proteins (lower panel) on chromosome I, II **(a)**, IV, and V **(b)**. Chromodomain protein tracks are shown in different colors. CHD family (green), proteins with no clear homolog in human (purple), HP1 family (yellow), and Msl-3 homolog family (red). The development stage of animals for each sample is noted as E (embryos), L (larval), and A (adult). **(c)** Percentage of chromodomain protein ChIP-seq peaks on chromosome arms and centers.

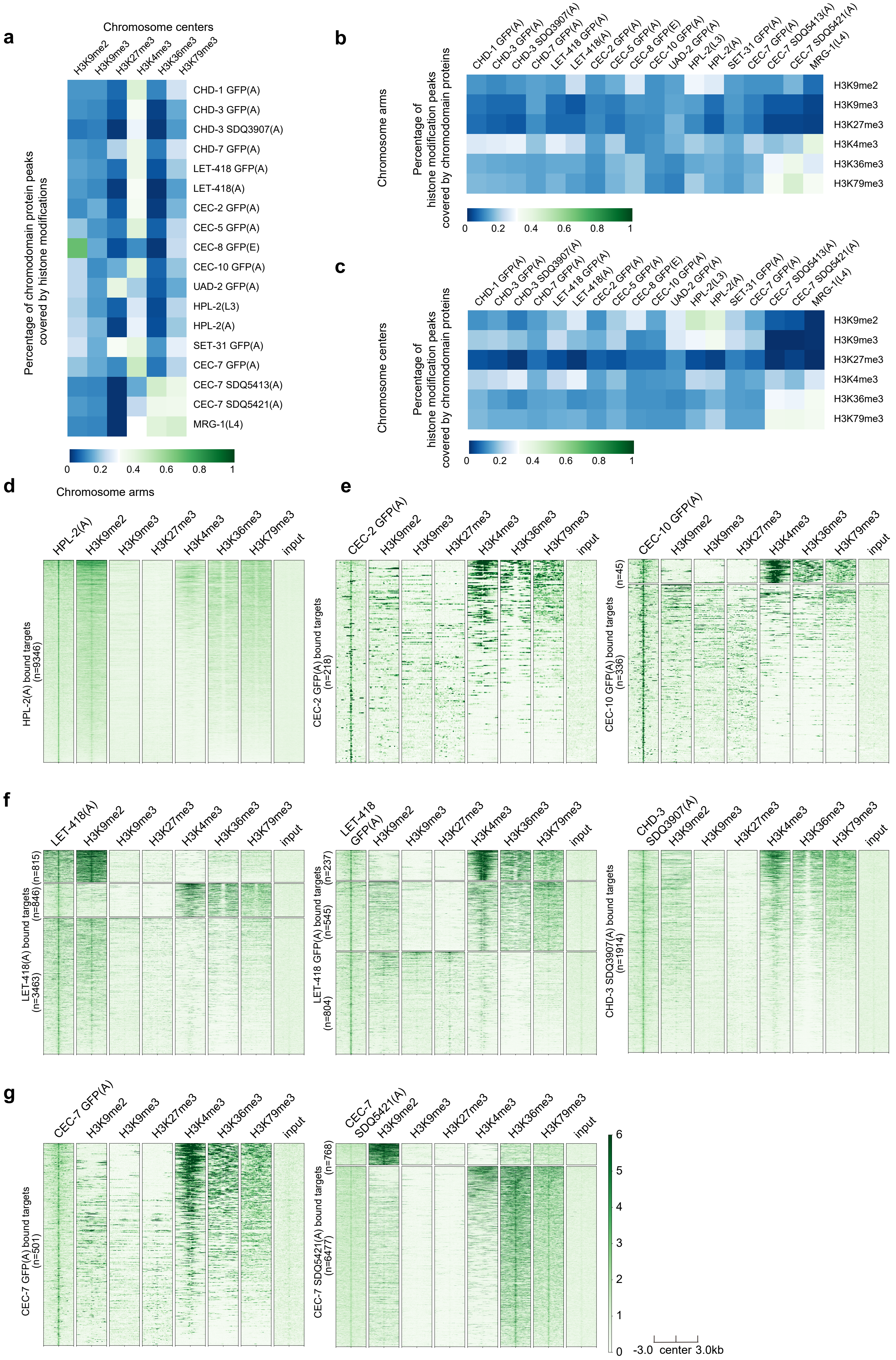
a
Chromosome centers
H3K9me2
H3K9me3
H3K27me3
H3K4me3
H3K36me3
H3K79me3
CHD-1 GFP(A)
CHD-3 GFP(A)
CHD-3 SDQ3907(A)
CHD-7 GFP(A)
LET-418 GFP(A)
LET-418(A)
CEC-2 GFP(A)
CEC-5 GFP(A)
CEC-8 GFP(E)
CEC-10 GFP(A)
UAD-2 GFP(A)
HPL-2(L3)
HPL-2(A)
SET-31 GFP(A)
CEC-7 GFP(A)
CEC-7 SDQ5413(A)
CEC-7 SDQ5421(A)
MRG-1(L4)
Percentage of chromodomain protein peaks covered by histone modifications
0
0.2
0.4
0.6
0.8
1
b
Chromosome arms
Percentage of histone modification peaks covered by chromodomain proteins
c
Chromosome centers
d
Chromosome arms
HPL-2(A)
input
HPL-2(A) bound targets (n=9346)
e
CEC-2 GFP(A) bound targets (n=218)
CEC-10 GFP(A) bound targets
(n=45)
(n=336)
f
LET-418(A) bound targets
(n=815)
(n=846)
(n=3463)
LET-418 GFP(A)
LET-418 GFP(A) bound targets
(n=237)
(n=545)
(n=804)
CHD-3 SDQ3907(A)
CHD-3 SDQ3907(A) bound targets (n=1914)
g
CEC-7 GFP(A) bound targets (n=501)
CEC-7 SDQ5421(A)
CEC-7 SDQ5421(A) bound targets
(n=768)
(n=6477)
6
5
4
3
2
1
0
-3.0
center
3.0kb

Supplementary Figure 8. Colocalization of chromodomain proteins with histone modifications identified by ChIP-seq assays. (a) Heatmap showing percentage of chromodomain protein peaks covered by histone modifications on chromosome centers. **(b, c)** Heatmaps showing percentage of histone modification peaks covered by chromodomain proteins on chromosome arms **(b)** and centers **(c)**. **(d, e, f, g)** Heatmaps comparing heterochromatin-enriched histone modifications (H3K9me2, H3K9me3, H3K27me3) and euchromatin-enriched histone modifications (H3K4me3, H3K36me3, H3K79me3) with the indicated chromodomain proteins on chromosome arms.

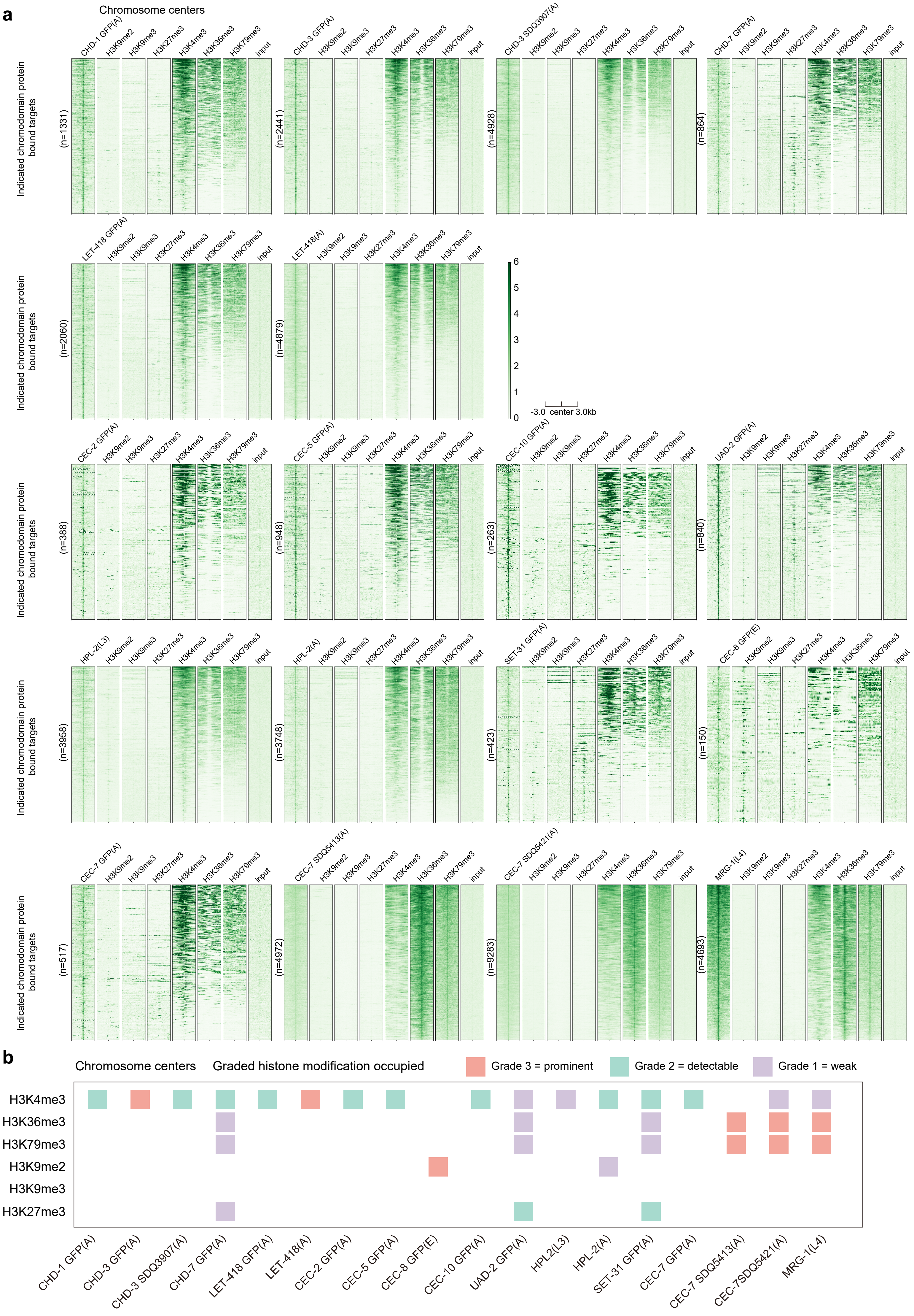
a
Chromosome centers
Indicated chromodomain protein bound targets
CHD-1 GFP(A)
CHD-3 GFP(A)
CHD-3 SDQ3907(A)
CHD-7 GFP(A)
LET-418 GFP(A)
LET-418(A)
CEC-2 GFP(A)
CEC-5 GFP(A)
CEC-10 GFP(A)
UAD-2 GFP(A)
HPL-2(L3)
HPL-2(A)
SET-31 GFP(A)
CEC-8 GFP(E)
CEC-7 GFP(A)
CEC-7 SDQ5413(A)
CEC-7 SDQ5421(A)
MRG-1(L4)
H3K9me2
H3K9me3
H3K27me3
H3K4me3
H3K36me3
H3K79me3
input
(n=1331)
(n=2441)
(n=4928)
(n=864)
(n=2060)
(n=4879)
(n=388)
(n=948)
(n=263)
(n=840)
(n=3958)
(n=3748)
(n=423)
(n=150)
(n=517)
(n=4972)
(n=9283)
(n=4693)
6
5
4
3
2
1
0
-3.0 center 3.0kb
b
Chromosome centers
Graded histone modification occupied
Grade 3 = prominent
Grade 2 = detectable
Grade 1 = weak
H3K4me3
H3K36me3
H3K79me3
H3K9me2
H3K9me3
H3K27me3
CHD-1 GFP(A)
CHD-3 GFP(A)
CHD-3 SDQ3907(A)
CHD-7 GFP(A)
LET-418 GFP(A)
LET-418(A)
CEC-2 GFP(A)
CEC-5 GFP(A)
CEC-8 GFP(E)
CEC-10 GFP(A)
UAD-2 GFP(A)
HPL2(L3)
HPL-2(A)
SET-31 GFP(A)
CEC-7 GFP(A)
CEC-7 SDQ5413(A)
CEC-7SDQ5421(A)
MRG-1(L4)

Supplementary Figure 9. Colocalization of chromodomain proteins with histone modifications identified by ChIP-seq assay on chromosome centers. (a) Heatmaps comparing heterochromatin-enriched histone modifications (H3K9me2, H3K9me3, H3K27me3) and euchromatin-enriched histone modifications (H3K4me3, H3K36me3, H3K79me3) with the indicated chromodomain proteins on chromosome centers. **(b)** A summary of the interaction of chromodomain proteins with histone modifications on chromosome centers. "Graded histone modification occupied" for a pair of chromodomain protein and histone modification fell into 3 categories: grade 3 = "prominent" (red), grade 2 = "detectable" (green), and grade 1 = "weak" (purple). Grade 3 was defined as pairs meeting all of the 3 criteria: 1. "Pearson correlation coefficient (r)≥0.300". 2. "≥30% of significant peaks occupied by a certain histone modification (using IntervalStats software package with P<0.05)". 3. "Overlap detected by heatmap analysis". Grade 2 meets 2 out of the 3 criteria and grade 1 meets any of the 3.

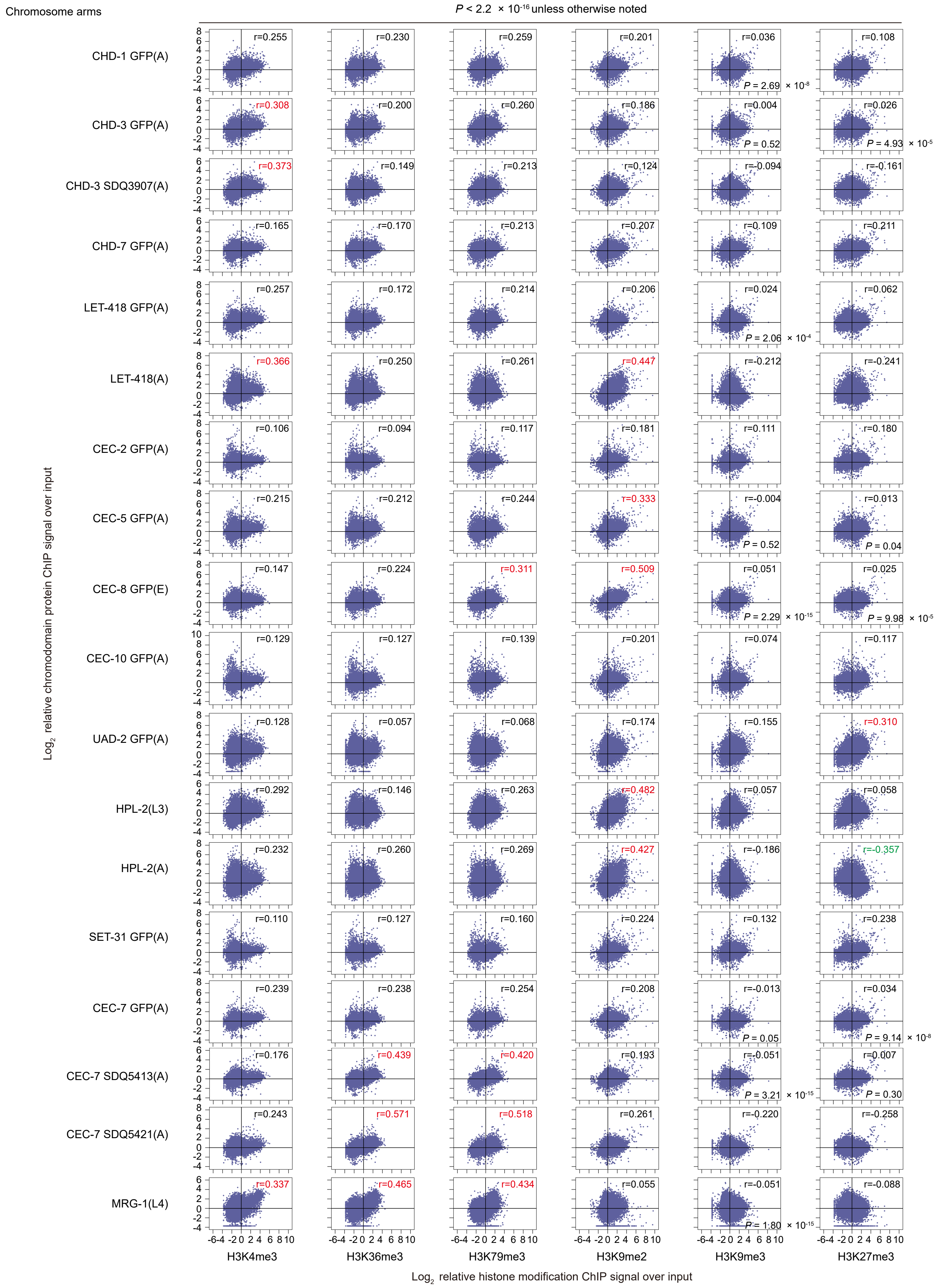

Chromosome arms
P < 2.2 × 10-16 unless otherwise noted
CHD-1 GFP(A)
CHD-3 GFP(A)
CHD-3 SDQ3907(A)
CHD-7 GFP(A)
LET-418 GFP(A)
LET-418(A)
CEC-2 GFP(A)
CEC-5 GFP(A)
CEC-8 GFP(E)
CEC-10 GFP(A)
UAD-2 GFP(A)
HPL-2(L3)
HPL-2(A)
SET-31 GFP(A)
CEC-7 GFP(A)
CEC-7 SDQ5413(A)
CEC-7 SDQ5421(A)
MRG-1(L4)
H3K4me3
H3K36me3
H3K79me3
H3K9me2
H3K9me3
H3K27me3
Log2 relative chromodomain protein ChIP signal over input
Log2 relative histone modification ChIP signal over input

Supplementary Figure 10. Colocalization of chromodomain proteins with histone modifications identified by ChIP-seq assays on chromosome arms. Scatter plots comparing $\log_2$ enrichments of indicated histone marks versus chromodomain proteins mapped by ChIP–seq. n = 24,284 fragments of genomic sites on chromosome arms (See Methods for details). A two-sided t-test was performed. $P < 2.2 \times 10^{-16}$ unless otherwise noted. r, Pearson correlation coefficient. $r \geq 0.300$ was colored by red and $r \leq -0.300$ was colored by green.

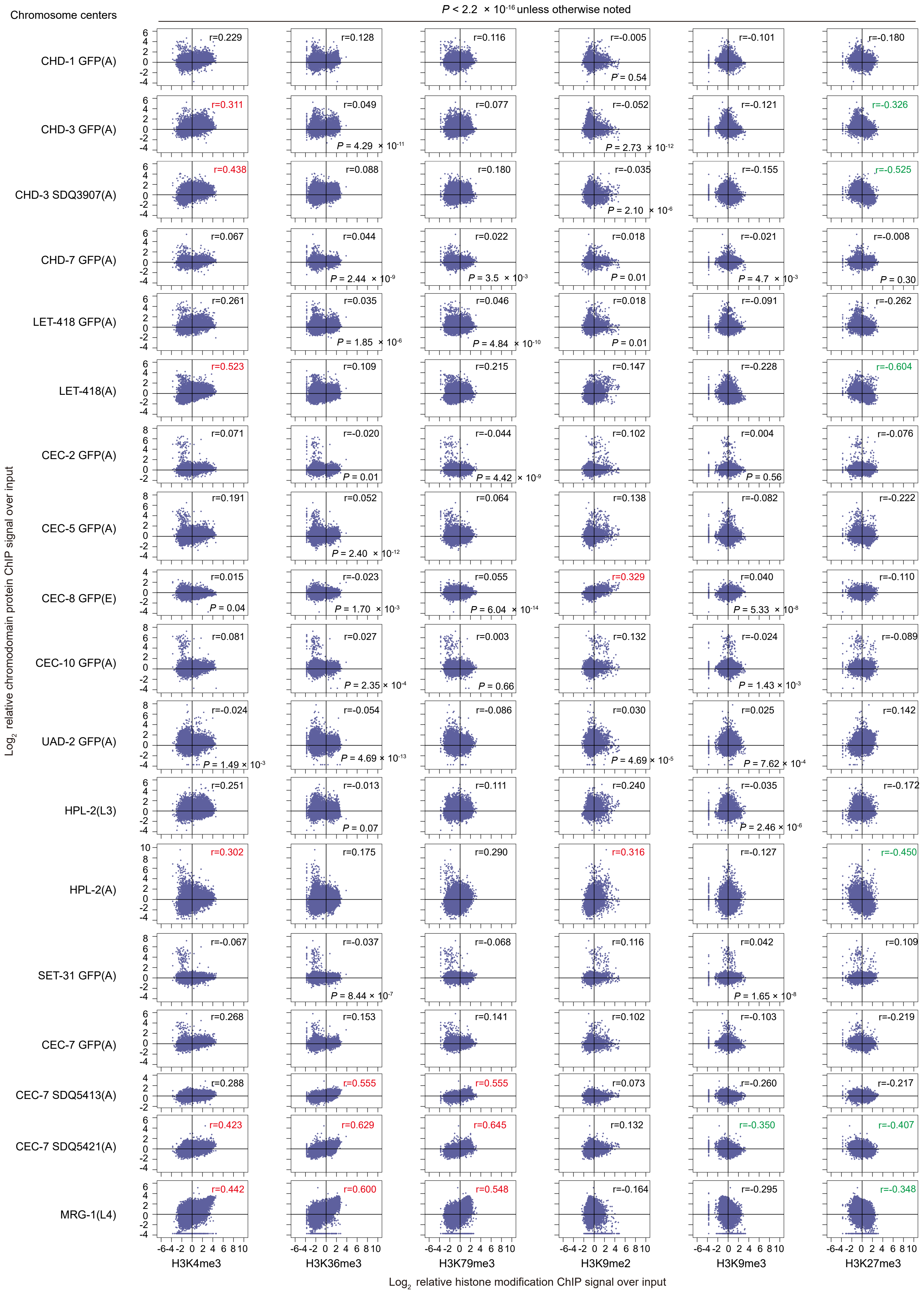
Chromosome centers
P < 2.2 × 10-16 unless otherwise noted
CHD-1 GFP(A)
CHD-3 GFP(A)
CHD-3 SDQ3907(A)
CHD-7 GFP(A)
LET-418 GFP(A)
LET-418(A)
CEC-2 GFP(A)
CEC-5 GFP(A)
CEC-8 GFP(E)
CEC-10 GFP(A)
UAD-2 GFP(A)
HPL-2(L3)
HPL-2(A)
SET-31 GFP(A)
CEC-7 GFP(A)
CEC-7 SDQ5413(A)
CEC-7 SDQ5421(A)
MRG-1(L4)
H3K4me3
H3K36me3
H3K79me3
H3K9me2
H3K9me3
H3K27me3
Log2 relative chromodomain protein ChIP signal over input
Log2 relative histone modification ChIP signal over input

Supplementary Figure 11. Colocalization of chromodomain proteins with histone modifications identified by ChIP-seq assays on chromosome centers. Scatter plots comparing log_2 enrichments of indicated histone marks versus chromodomain proteins mapped by ChIP–seq. n=18,212 fragments of genomic sites on chromosome centers (See Methods for details). A two-sided t-test was performed. $P < 2.2 \times 10^{-16}$ unless otherwise noted. r, Pearson correlation coefficient. $r \geq 0.300$ was colored by red and $r \leq -0.300$ was colored by green.

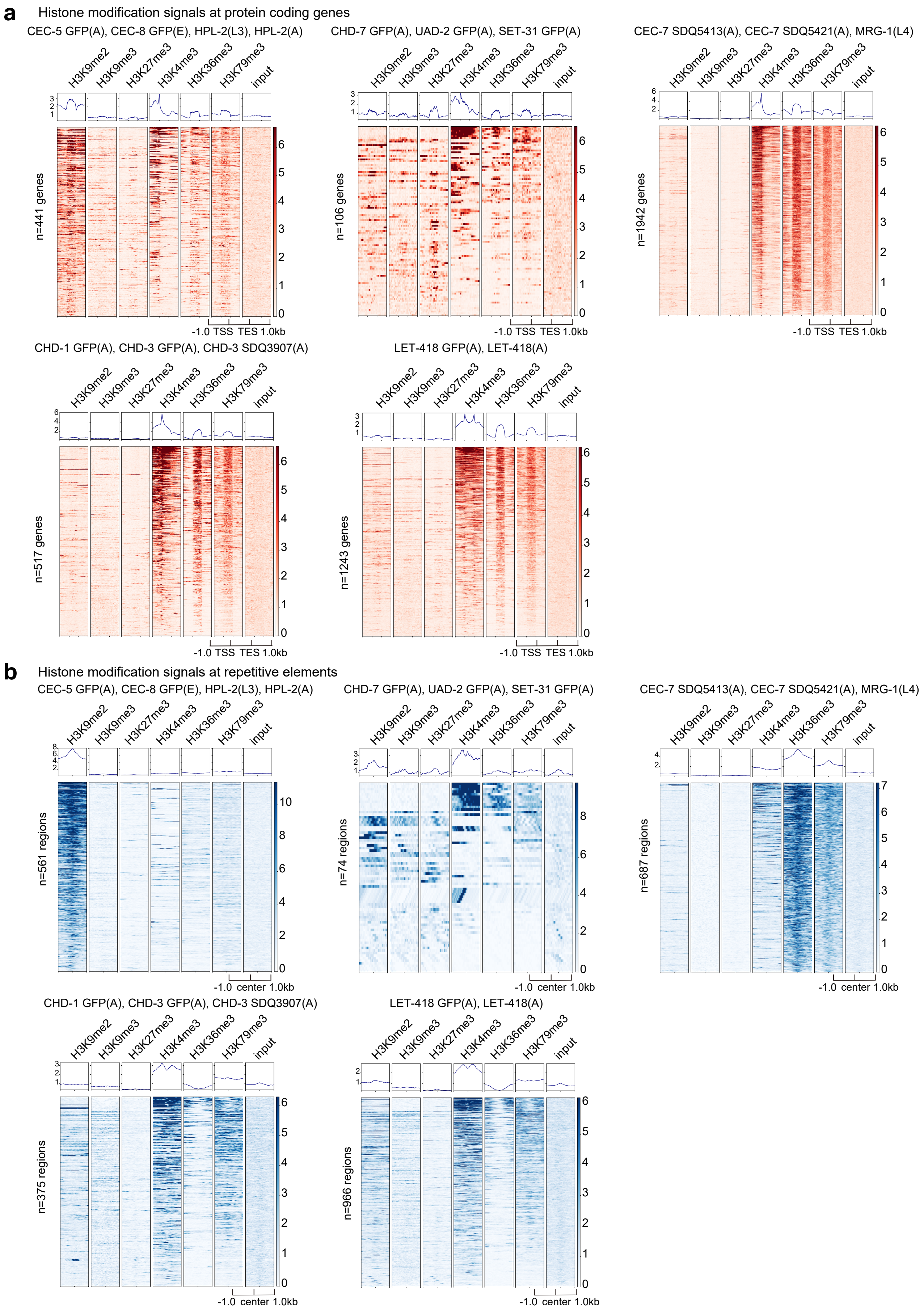

Supplementary Figure 12. Different combination of chromodomain protein binding sites were covered by distinct histone modifications. (a, b) Profile plots and heatmaps showing heterochromatin-enriched histone modifications (H3K9me2, H3K9me3, H3K27me3) and euchromatin-enriched histone modifications (H3K4me3, H3K36me3, H3K79me3) coverage on the overlapped binding sites (protein coding genes **(a)**, repetitive elements **(b)**) of indicated chromodomain proteins. Each horizontal line shows an overlapped peak of indicated chromodomain proteins. n, number of peaks. Raw reads were normalized to bins per million mapped reads (BPM). The color scale shows the range of heatmap intensities.

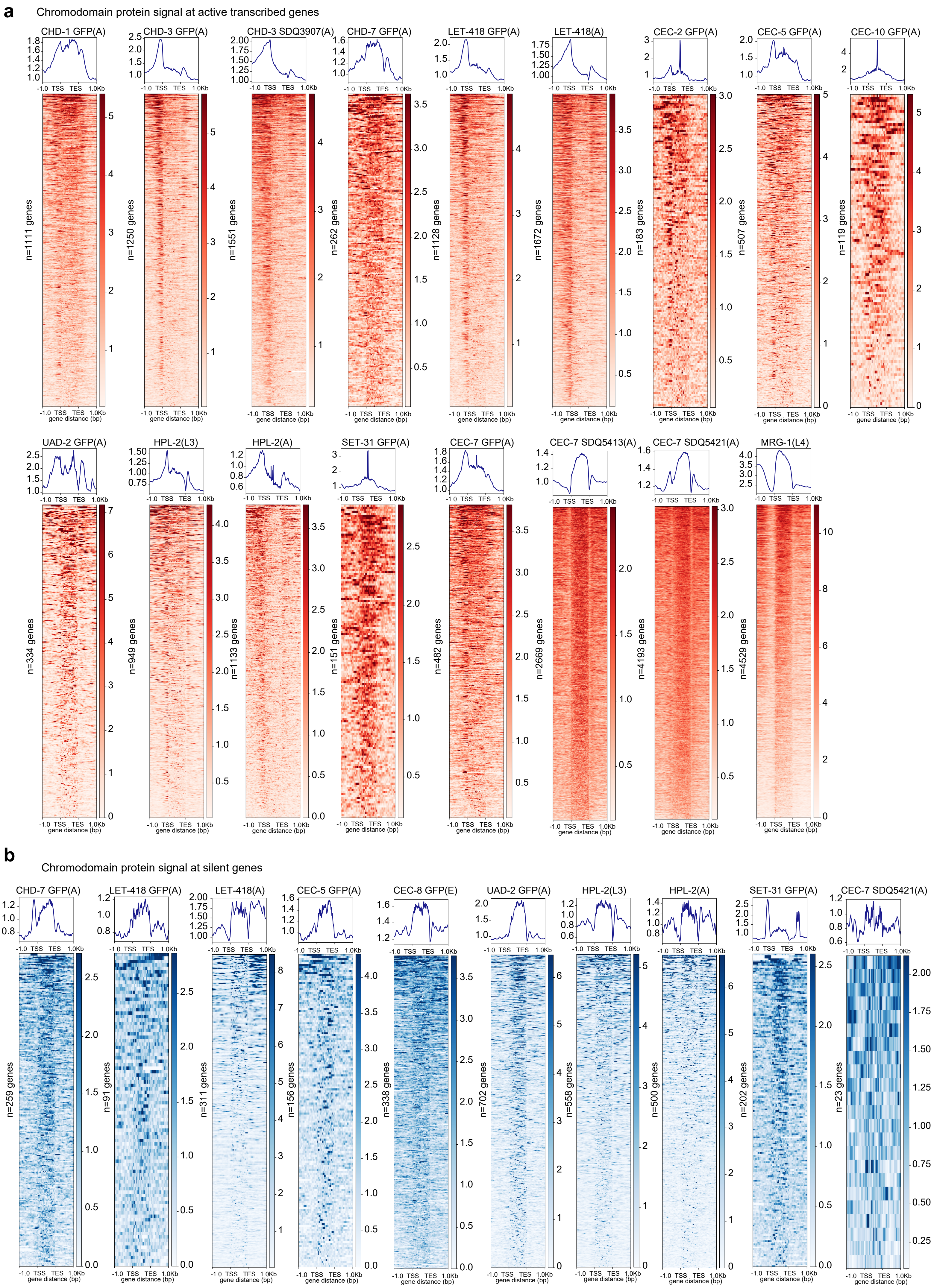
a
Chromodomain protein signal at active transcribed genes
CHD-1 GFP(A)
CHD-3 GFP(A)
CHD-3 SDQ3907(A)
CHD-7 GFP(A)
LET-418 GFP(A)
LET-418(A)
CEC-2 GFP(A)
CEC-5 GFP(A)
CEC-10 GFP(A)
n=1111 genes
n=1250 genes
n=1551 genes
n=262 genes
n=1128 genes
n=1672 genes
n=183 genes
n=507 genes
n=119 genes
UAD-2 GFP(A)
HPL-2(L3)
HPL-2(A)
SET-31 GFP(A)
CEC-7 GFP(A)
CEC-7 SDQ5413(A)
CEC-7 SDQ5421(A)
MRG-1(L4)
n=334 genes
n=949 genes
n=1133 genes
n=151 genes
n=482 genes
n=2669 genes
n=4193 genes
n=4529 genes
-1.0 TSS TES 1.0Kb
gene distance (bp)
b
Chromodomain protein signal at silent genes
CHD-7 GFP(A)
LET-418 GFP(A)
LET-418(A)
CEC-5 GFP(A)
CEC-8 GFP(E)
UAD-2 GFP(A)
HPL-2(L3)
HPL-2(A)
SET-31 GFP(A)
CEC-7 SDQ5421(A)
n=259 genes
n=91 genes
n=311 genes
n=156 genes
n=338 genes
n=702 genes
n=558 genes
n=500 genes
n=202 genes
n=23 genes

Supplementary Figure 13. Distribution of indicated chromodomain proteins across target genes.
(a, b) Profile plots and heatmaps showing chromodomain protein ChIP-seq signals around transcription start sites (TSS) and transcript end sites (TES) of actively transcribed **(a)** and silent targets **(b)**. Each horizontal line shows a chromodomain protein target gene. n, number of target genes. Raw reads were normalized to bins per million mapped reads (BPM). The color scale shows the range of heatmap intensities.

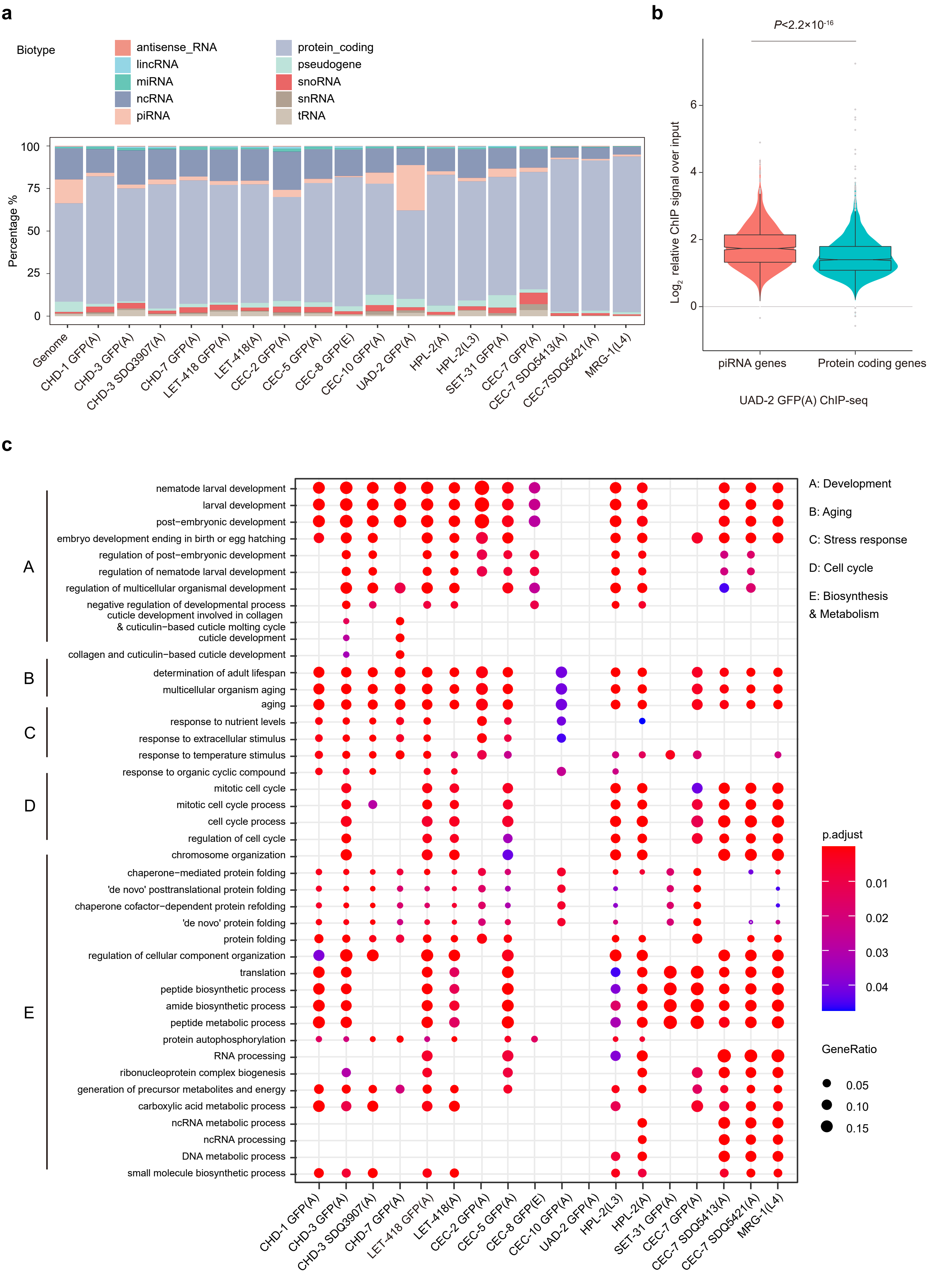

a
Biotype
antisense_RNA
lincRNA
miRNA
ncRNA
piRNA
protein_coding
pseudogene
snoRNA
snRNA
tRNA
Percentage %
Genome
CHD-1 GFP(A)
CHD-3 GFP(A)
CHD-3 SDQ3907(A)
CHD-7 GFP(A)
LET-418 GFP(A)
LET-418(A)
CEC-2 GFP(A)
CEC-5 GFP(A)
CEC-8 GFP(E)
CEC-10 GFP(A)
UAD-2 GFP(A)
HPL-2(A)
HPL-2(L3)
SET-31 GFP(A)
CEC-7 GFP(A)
CEC-7 SDQ5413(A)
CEC-7SDQ5421(A)
MRG-1(L4)
b
P<2.2×10-16
Log2 relative ChIP signal over input
piRNA genes
Protein coding genes
UAD-2 GFP(A) ChIP-seq
c
A
B
C
D
E
nematode larval development
larval development
post−embryonic development
embryo development ending in birth or egg hatching
regulation of post−embryonic development
regulation of nematode larval development
regulation of multicellular organismal development
negative regulation of developmental process
cuticle development involved in collagen & cuticulin−based cuticle molting cycle
cuticle development
collagen and cuticulin−based cuticle development
determination of adult lifespan
multicellular organism aging
aging
response to nutrient levels
response to extracellular stimulus
response to temperature stimulus
response to organic cyclic compound
mitotic cell cycle
mitotic cell cycle process
cell cycle process
regulation of cell cycle
chromosome organization
chaperone−mediated protein folding
'de novo' posttranslational protein folding
chaperone cofactor−dependent protein refolding
'de novo' protein folding
protein folding
regulation of cellular component organization
translation
peptide biosynthetic process
amide biosynthetic process
peptide metabolic process
protein autophosphorylation
RNA processing
ribonucleoprotein complex biogenesis
generation of precursor metabolites and energy
carboxylic acid metabolic process
ncRNA metabolic process
ncRNA processing
DNA metabolic process
small molecule biosynthetic process
A: Development
B: Aging
C: Stress response
D: Cell cycle
E: Biosynthesis & Metabolism
p.adjust
GeneRatio
CHD-1 GFP(A)
CHD-3 GFP(A)
CHD-3 SDQ3907(A)
CHD-7 GFP(A)
LET-418 GFP(A)
LET-418(A)
CEC-2 GFP(A)
CEC-5 GFP(A)
CEC-8 GFP(E)
CEC-10 GFP(A)
UAD-2 GFP(A)
HPL-2(L3)
HPL-2(A)
SET-31 GFP(A)
CEC-7 GFP(A)
CEC-7 SDQ5413(A)
CEC-7 SDQ5421(A)
MRG-1(L4)

Supplementary Figure 14. The chromodomain proteins mainly bound protein-coding genes. (a) Gene biotypes bound by chromodomain proteins. **(b)** Boxplot showing $\log_2$ UAD-2 ChIP-seq signals over input on piRNA (n=960) and protein coding gene (n=1856) targets. Upper and lower adjacent values are shown by whiskers. 75th and 25th percentiles are shown by the bounds of box. 50th percentile is shown by the center of box. Significance was tested with one-sided unpaired Wilcoxon test, $p < 2.2 \times 10^{-16}$. **(c)** GO term analysis of chromodomain protein targets. The area of the bubble reveals the number of genes belonging to the indicated GO categories. Color indicates statistical significance, ranging from highly significant enrichment (red) to enrichment with lower significance (blue). Significance was tested with one-sided Fisher's exact test. P value was adjusted using FDR. Significantly enriched GO terms were selected based on an adjusted P value< 0.05.

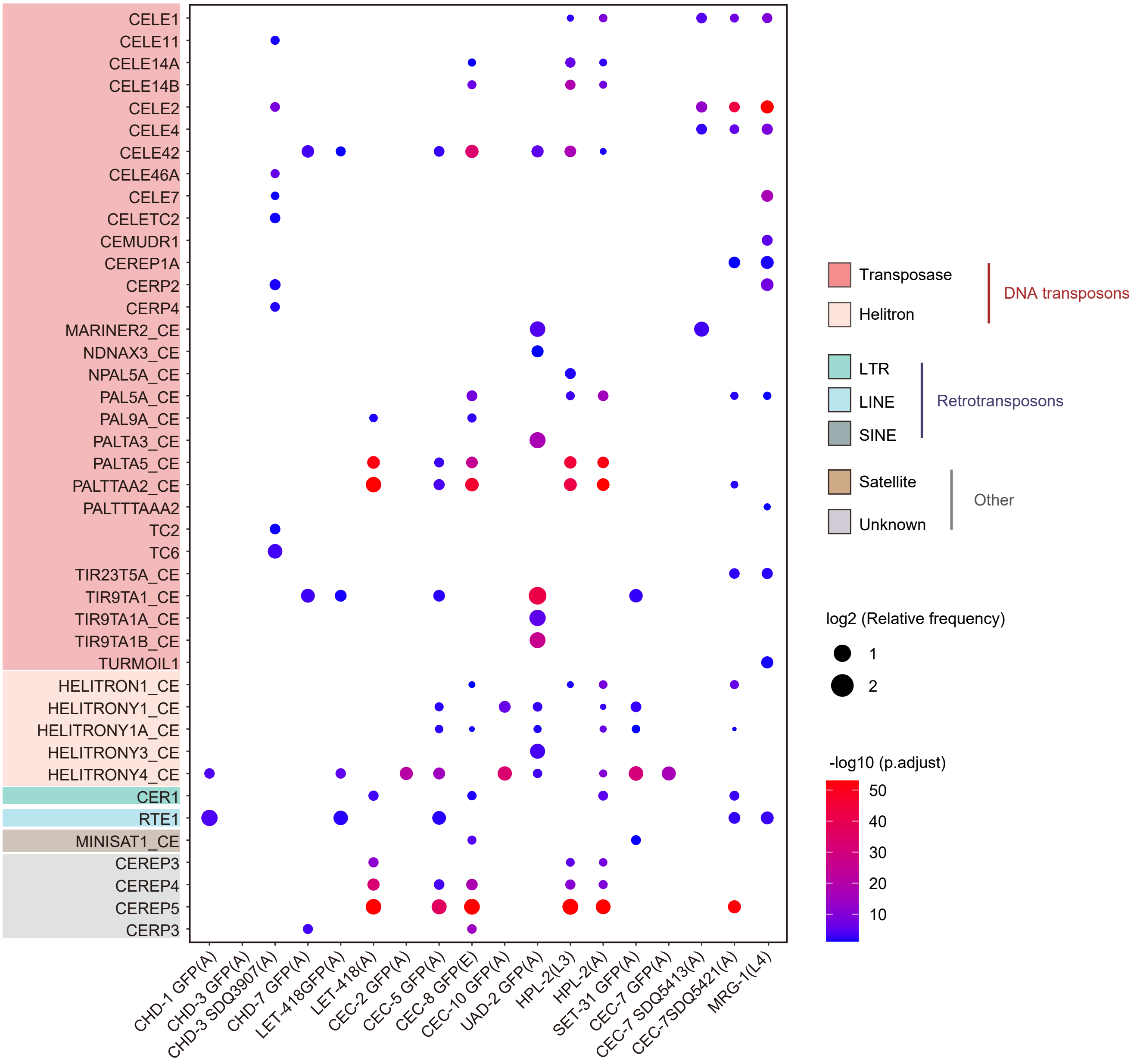

Supplementary Figure 15. The chromodomain proteins were enriched on transposons and Helitron repetitive sequence families. Enrichment analysis of chromodomain proteins in repetitive sequence families. The area of the bubble reveals the relative frequency of repetitive sequences belonging to the indicated families. Colors indicate statistical significance, ranging from highly significant enrichment (red) to less significant enrichment (blue). Significance was tested with one-sided Fisher's exact test. P value was adjusted using "BH". Significantly enriched repetitive sequences were selected based on an adjusted P value< 0.05.

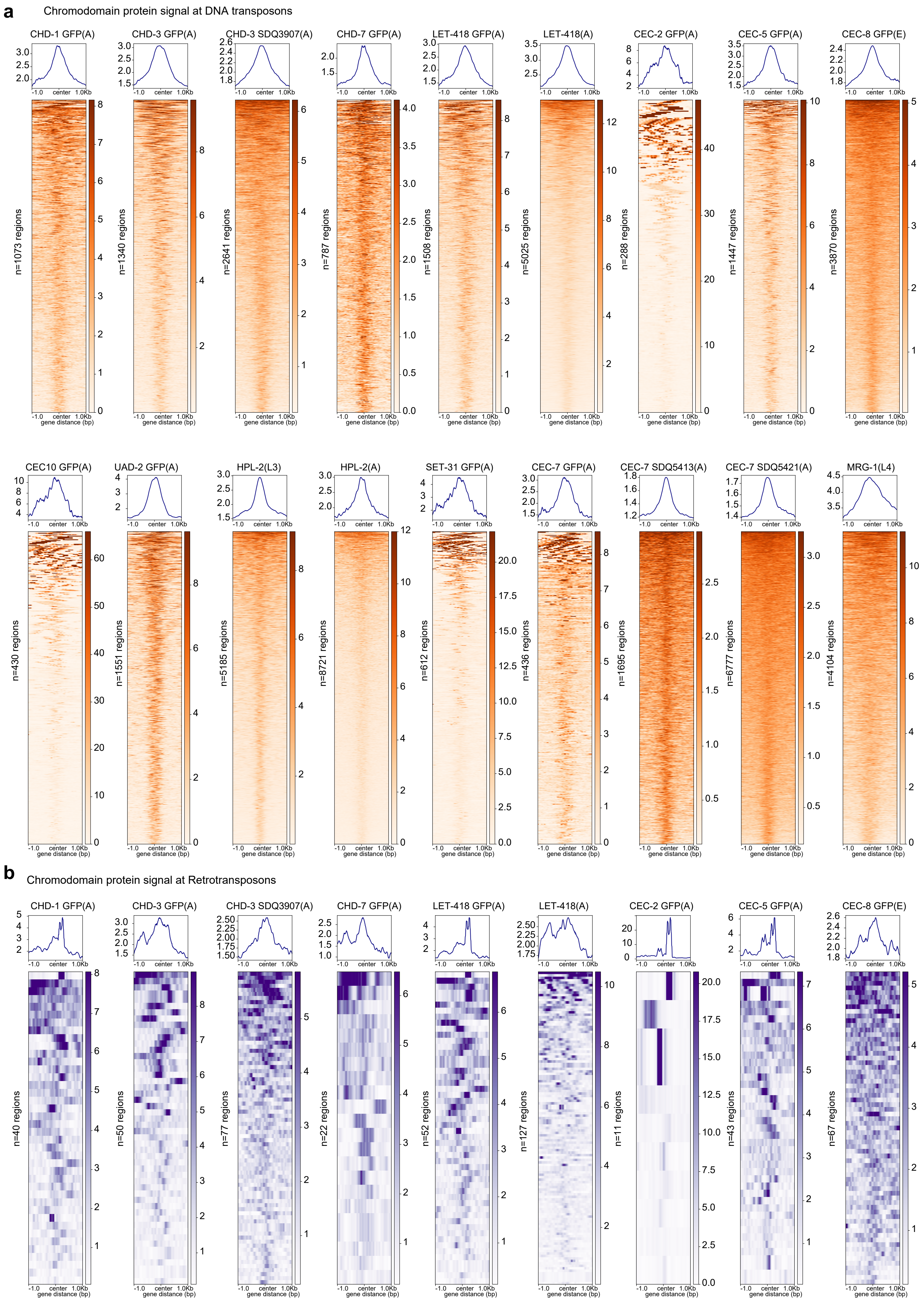
a
Chromodomain protein signal at DNA transposons
CHD-1 GFP(A)
CHD-3 GFP(A)
CHD-3 SDQ3907(A)
CHD-7 GFP(A)
LET-418 GFP(A)
LET-418(A)
CEC-2 GFP(A)
CEC-5 GFP(A)
CEC-8 GFP(E)
n=1073 regions
n=1340 regions
n=2641 regions
n=787 regions
n=1508 regions
n=5025 regions
n=288 regions
n=1447 regions
n=3870 regions
CEC10 GFP(A)
UAD-2 GFP(A)
HPL-2(L3)
HPL-2(A)
SET-31 GFP(A)
CEC-7 GFP(A)
CEC-7 SDQ5413(A)
CEC-7 SDQ5421(A)
MRG-1(L4)
n=430 regions
n=1551 regions
n=5185 regions
n=8721 regions
n=612 regions
n=436 regions
n=1695 regions
n=6777 regions
n=4104 regions
-1.0
center
1.0Kb
gene distance (bp)
b
Chromodomain protein signal at Retrotransposons
CHD-1 GFP(A)
CHD-3 GFP(A)
CHD-3 SDQ3907(A)
CHD-7 GFP(A)
LET-418 GFP(A)
LET-418(A)
CEC-2 GFP(A)
CEC-5 GFP(A)
CEC-8 GFP(E)
n=40 regions
n=50 regions
n=77 regions
n=22 regions
n=52 regions
n=127 regions
n=11 regions
n=43 regions
n=67 regions

Chromodomain protein signal at Retrotransposons

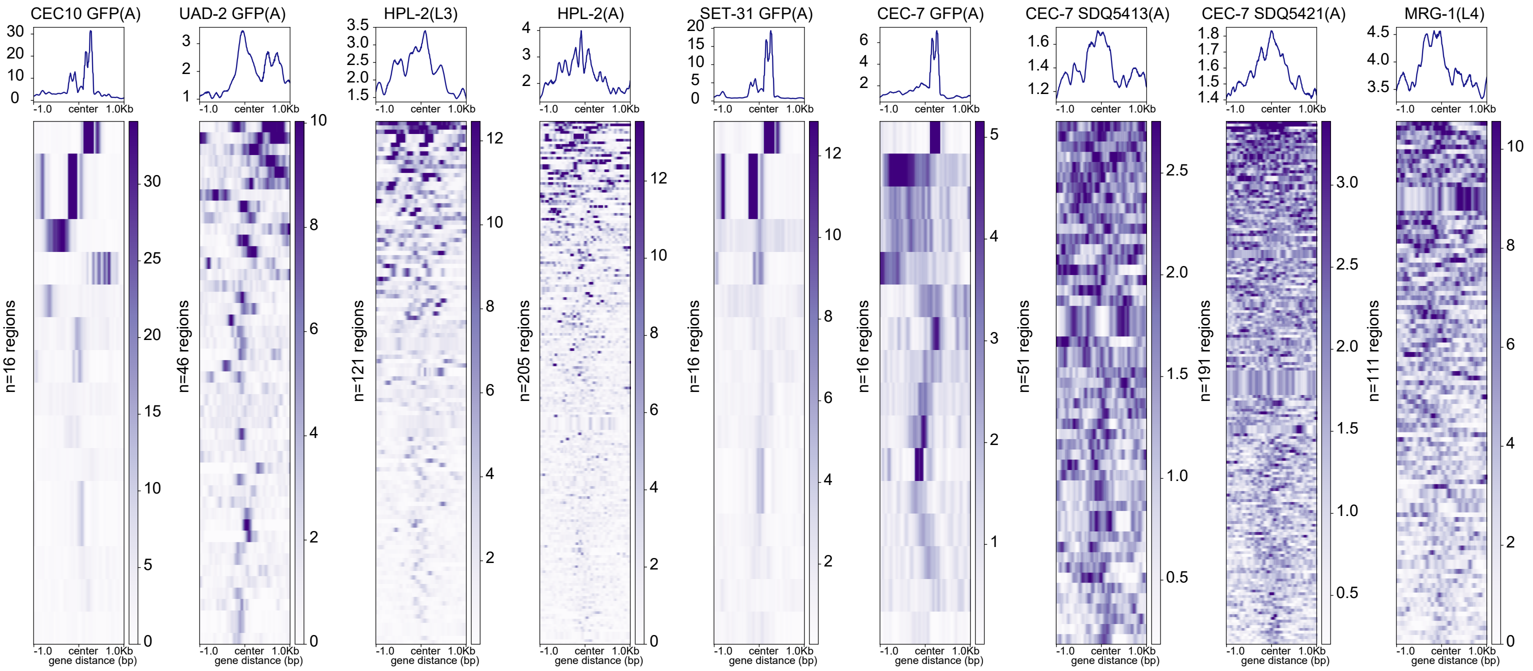

c Chromodomain protein signal at other repetitive elements

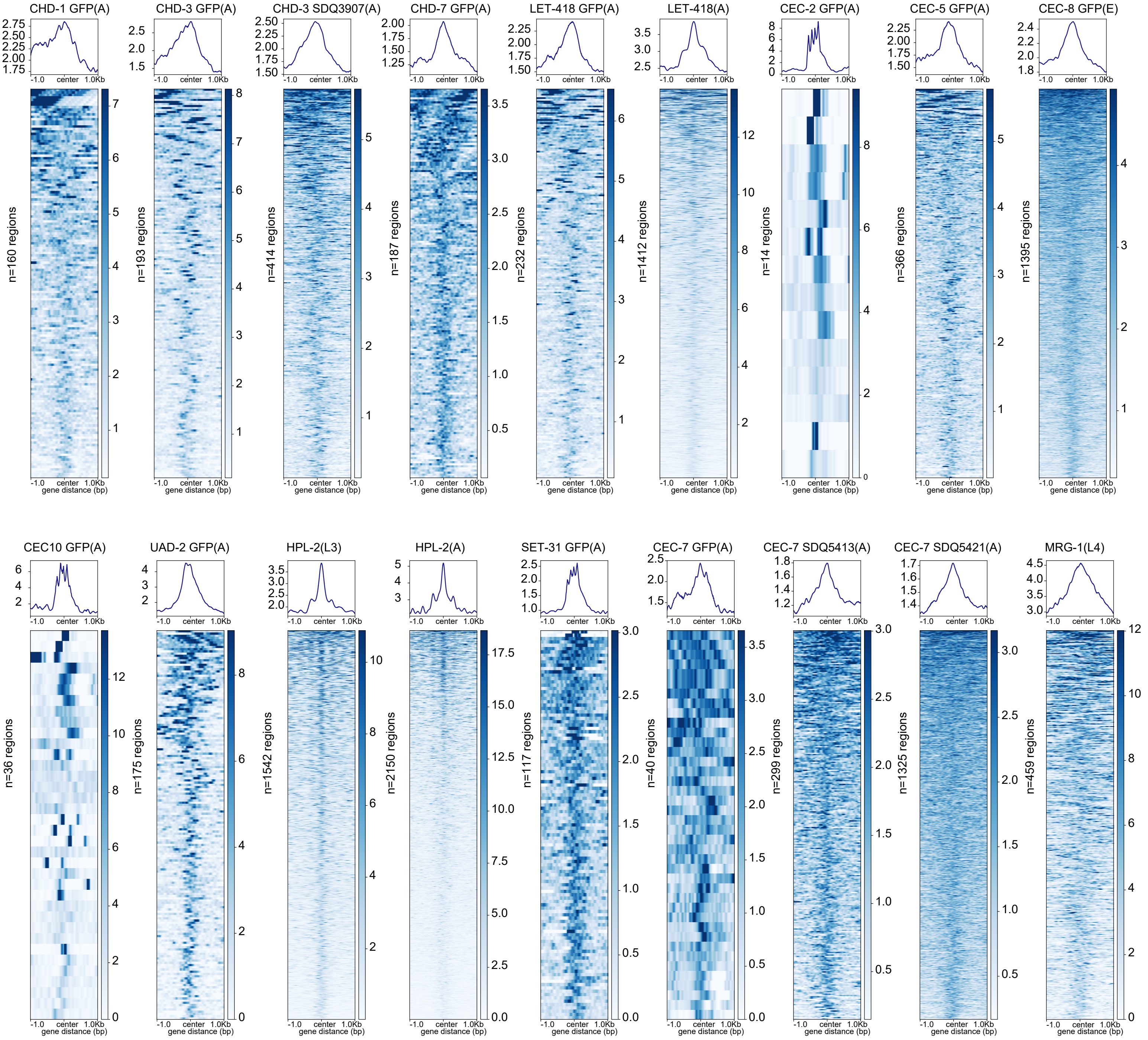

Supplementary Figure 16. Distribution of indicated chromodomain proteins across repetitive elements. **(a, b, c)** Profile plots and heatmaps showing chromodomain protein ChIP-seq signals at DNA transposons **(a)**, Retrotransposons **(b)** and other repetitive elements **(c)**. Each horizontal line shows a chromodomain protein bound repetitive element. n, number of repetitive elements. Raw reads were normalized to bins per million mapped reads (BPM). The color scale shows the range of heatmap intensities.

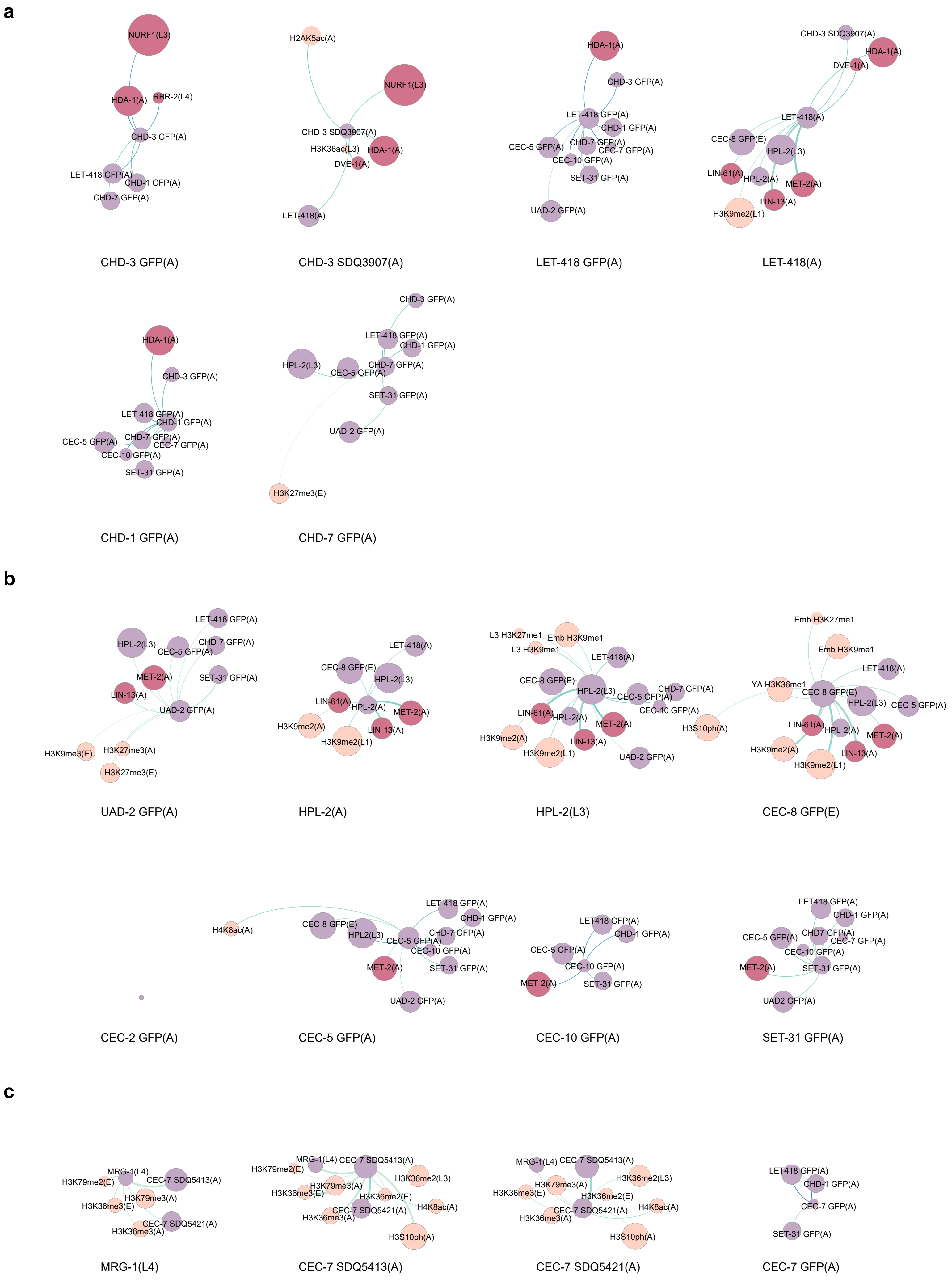
a
NURF1(L3)
HDA-1(A)
RBR-2(L4)
CHD-3 GFP(A)
LET-418 GFP(A)
CHD-1 GFP(A)
CHD-7 GFP(A)
CHD-3 GFP(A)
H2AK5ac(A)
NURF1(L3)
CHD-3 SDQ3907(A)
H3K36ac(L3)
HDA-1(A)
DVE-1(A)
LET-418(A)
CHD-3 SDQ3907(A)
HDA-1(A)
CHD-3 GFP(A)
LET-418 GFP(A)
CHD-1 GFP(A)
CEC-5 GFP(A)
CHD-7 GFP(A)
CEC-7 GFP(A)
CEC-10 GFP(A)
SET-31 GFP(A)
UAD-2 GFP(A)
LET-418 GFP(A)
CHD-3 SDQ3907(A)
HDA-1(A)
DVE-1(A)
LET-418(A)
CEC-8 GFP(E)
HPL-2(L3)
LIN-61(A)
HPL-2(A)
MET-2(A)
LIN-13(A)
H3K9me2(L1)
LET-418(A)
HDA-1(A)
CHD-3 GFP(A)
LET-418 GFP(A)
CHD-1 GFP(A)
CEC-5 GFP(A)
CHD-7 GFP(A)
CEC-7 GFP(A)
CEC-10 GFP(A)
SET-31 GFP(A)
CHD-1 GFP(A)
CHD-3 GFP(A)
LET-418 GFP(A)
CHD-1 GFP(A)
HPL-2(L3)
CEC-5 GFP(A)
CHD-7 GFP(A)
SET-31 GFP(A)
UAD-2 GFP(A)
H3K27me3(E)
CHD-7 GFP(A)
b
LET-418 GFP(A)
HPL-2(L3)
CEC-5 GFP(A)
CHD-7 GFP(A)
MET-2(A)
SET-31 GFP(A)
LIN-13(A)
UAD-2 GFP(A)
H3K9me3(E)
H3K27me3(A)
H3K27me3(E)
UAD-2 GFP(A)
LET-418(A)
CEC-8 GFP(E)
HPL-2(L3)
LIN-61(A)
HPL-2(A)
MET-2(A)
H3K9me2(A)
LIN-13(A)
H3K9me2(L1)
HPL-2(A)
L3 H3K27me1
Emb H3K9me1
L3 H3K9me1
LET-418(A)
CEC-8 GFP(E)
HPL-2(L3)
CHD-7 GFP(A)
CEC-5 GFP(A)
CEC-10 GFP(A)
LIN-61(A)
HPL-2(A)
MET-2(A)
H3S10ph(A)
H3K9me2(A)
LIN-13(A)
H3K9me2(L1)
UAD-2 GFP(A)
HPL-2(L3)
Emb H3K27me1
Emb H3K9me1
LET-418(A)
YA H3K36me1
CEC-8 GFP(E)
HPL-2(L3)
CEC-5 GFP(A)
LIN-61(A)
HPL-2(A)
MET-2(A)
H3K9me2(A)
LIN-13(A)
H3K9me2(L1)
CEC-8 GFP(E)
LET-418 GFP(A)
H4K8ac(A)
CHD-1 GFP(A)
CEC-8 GFP(E)
HPL2(L3)
CEC-5 GFP(A)
CHD-7 GFP(A)
CEC-10 GFP(A)
MET-2(A)
SET-31 GFP(A)
UAD-2 GFP(A)
LET418 GFP(A)
CHD-1 GFP(A)
CEC-5 GFP(A)
CEC-10 GFP(A)
MET-2(A)
SET-31 GFP(A)
LET418 GFP(A)
CHD-1 GFP(A)
CEC-5 GFP(A)
CHD7 GFP(A)
CEC-7 GFP(A)
CEC-10 GFP(A)
MET-2(A)
SET-31 GFP(A)
UAD2 GFP(A)
CEC-2 GFP(A)
CEC-5 GFP(A)
CEC-10 GFP(A)
SET-31 GFP(A)
c
MRG-1(L4)
H3K79me2(E)
CEC-7 SDQ5413(A)
H3K79me3(A)
H3K36me3(E)
CEC-7 SDQ5421(A)
H3K36me3(A)
MRG-1(L4)
CEC-7 SDQ5413(A)
H3K79me2(E)
H3K36me2(L3)
H3K79me3(A)
H3K36me3(E)
H3K36me2(E)
CEC-7 SDQ5421(A)
H4K8ac(A)
H3K36me3(A)
H3S10ph(A)
MRG-1(L4)
CEC-7 SDQ5413(A)
H3K36me2(L3)
H3K79me3(A)
H3K36me3(E)
H3K36me2(E)
CEC-7 SDQ5421(A)
H4K8ac(A)
H3K36me3(A)
H3S10ph(A)
LET418 GFP(A)
CHD-1 GFP(A)
CEC-7 GFP(A)
SET-31 GFP(A)
MRG-1(L4)
CEC-7 SDQ5413(A)
CEC-7 SDQ5421(A)
CEC-7 GFP(A)

Supplementary Figure 17. Chromodomain proteins colocalized with histone modifications and other epigenetic regulators. (a, b, c) The subnetworks showing colocalized factors of the indicated chromodomain proteins. In the subnetworks, nodes indicate individual chromodomain proteins (purple), histone modifications (light pink) and other epigenetic regulators (dark pink); edge colors depict the percentages of overlap between the nodes and weight the colocalization specificity between two factors (see Methods for details). The development stage of animals for each sample is noted as E (embryos), L (larval), and A (adult).

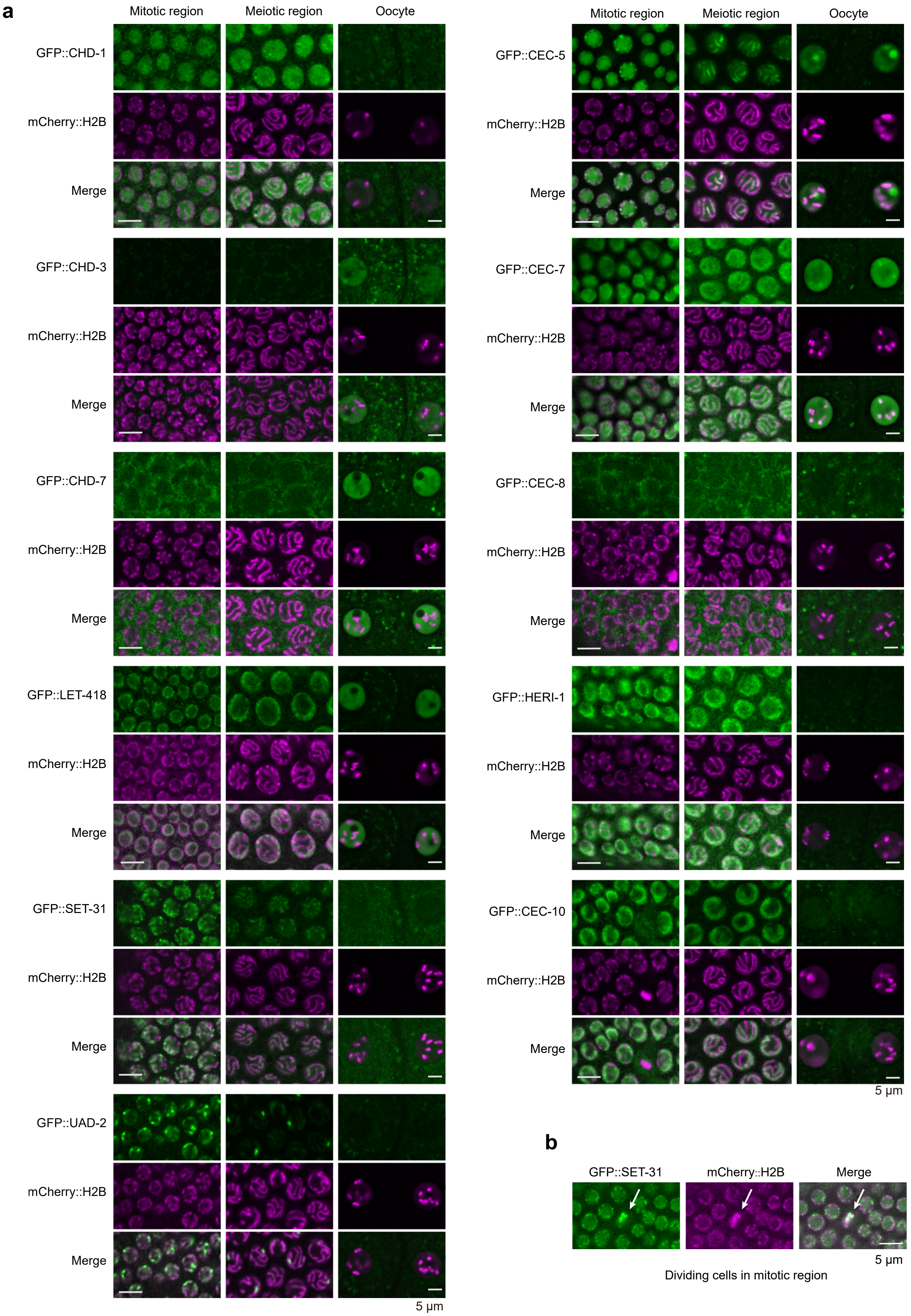

Supplementary Figure 18. The subcellular localization of chromodomain proteins in germline. (a) The localization of representative images for each chromodomain protein in mitotic cells, meiotic cells, and oocytes are shown (scale bar, 5 μm). The chromosome is marked by mCherry::H2B. **(b)** SET-31 colocalized with mCherry::H2B in dividing cells in mitotic zones of germline (indicated by white arrows). The chromosome is marked by mCherry::H2B.

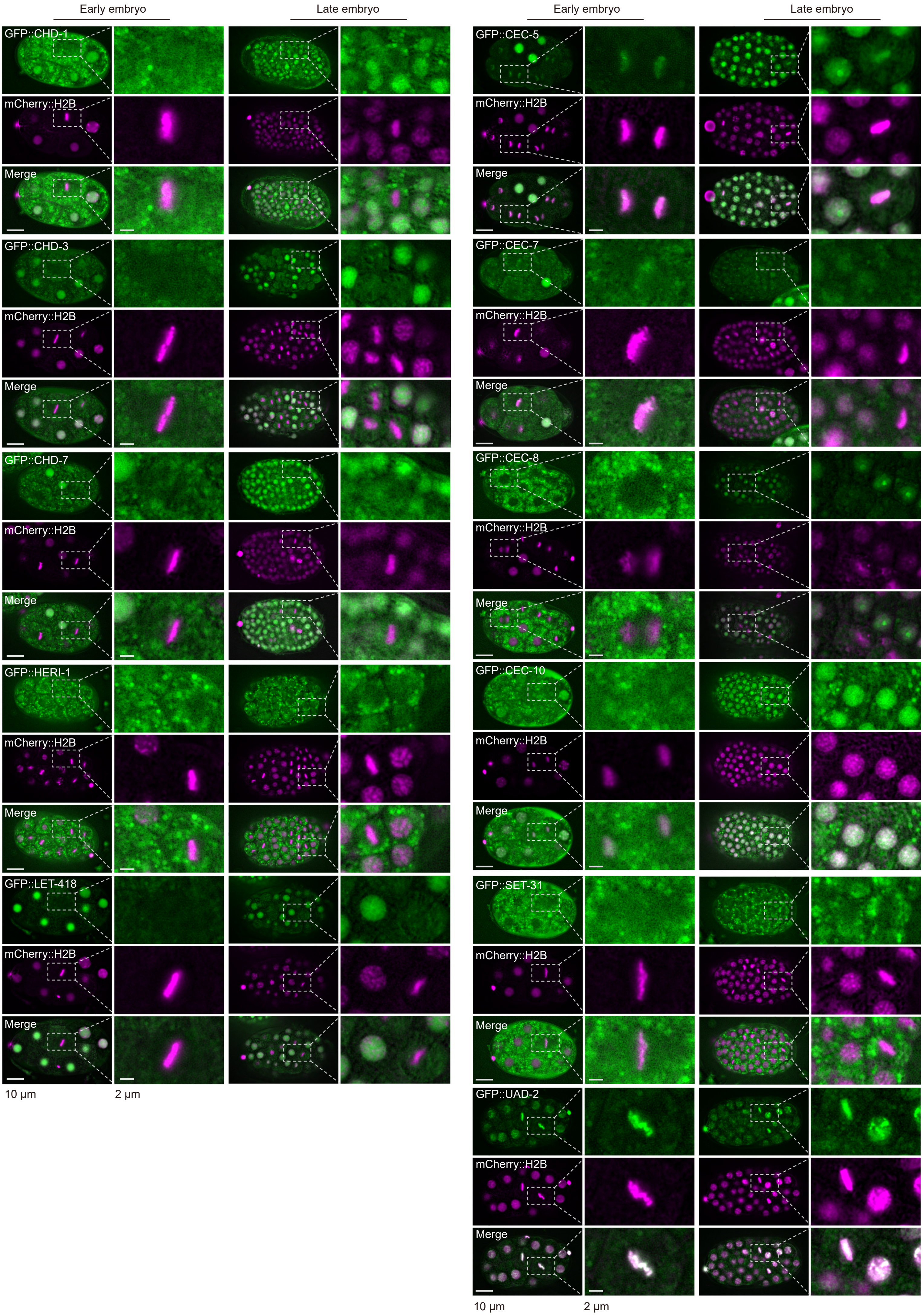

Early embryo
Late embryo
GFP::CHD-1
mCherry::H2B
Merge
GFP::CHD-3
mCherry::H2B
Merge
GFP::CHD-7
mCherry::H2B
Merge
GFP::HERI-1
mCherry::H2B
Merge
GFP::LET-418
mCherry::H2B
Merge
10 µm
2 µm
Early embryo
Late embryo
GFP::CEC-5
mCherry::H2B
Merge
GFP::CEC-7
mCherry::H2B
Merge
GFP::CEC-8
mCherry::H2B
Merge
GFP::CEC-10
mCherry::H2B
Merge
GFP::SET-31
mCherry::H2B
Merge
GFP::UAD-2
mCherry::H2B
Merge
10 µm
2 µm

Supplementary Figure 19. The subcellular localization of chromodomain proteins in embryos.
The localization of representative images for each chromodomain protein in early embryo and late embryo are shown (scale bar for overview images, 10 μm; scale bar for zoom in images, 2 μm). The chromosome is marked by mCherry::H2B.

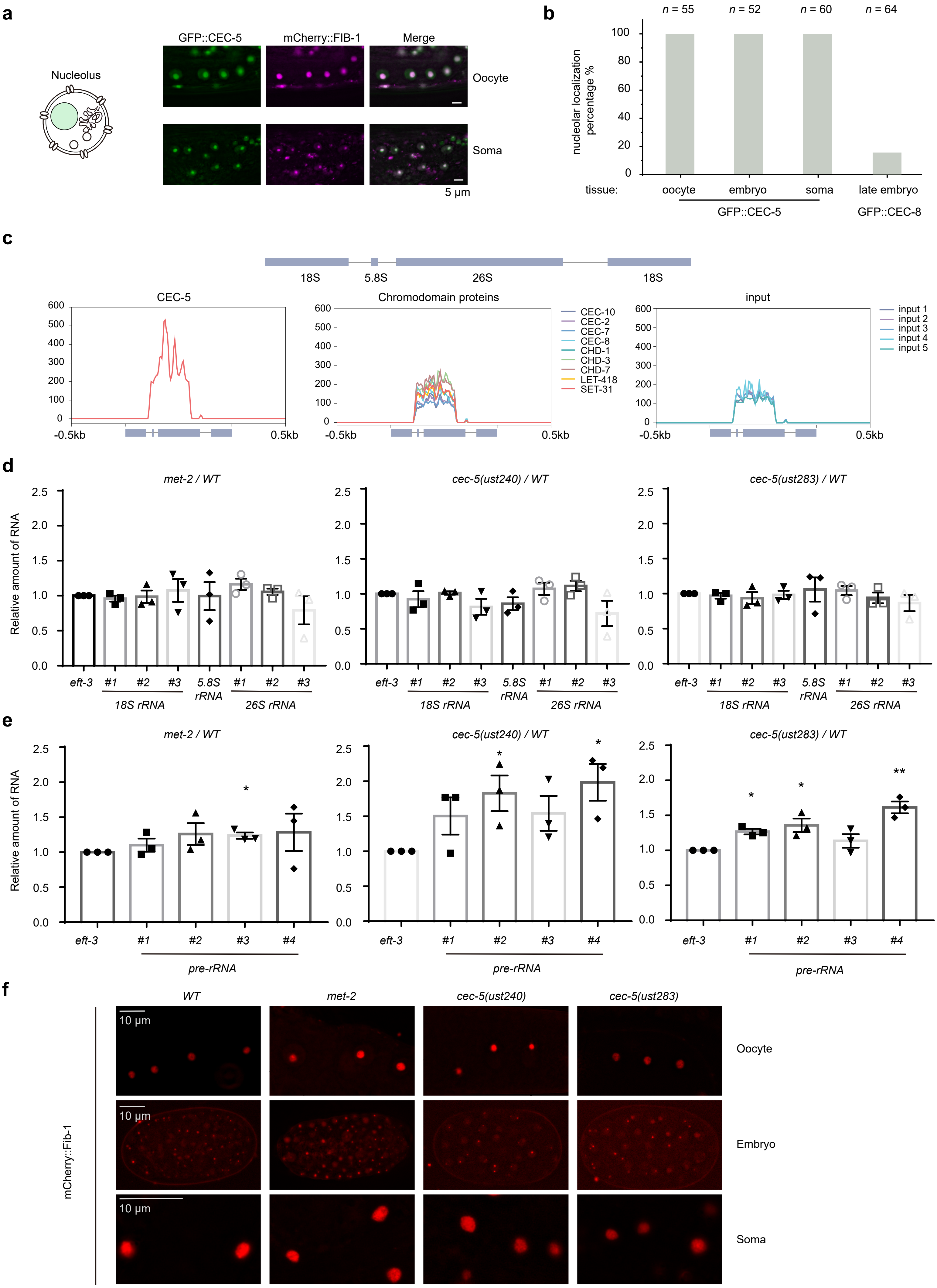

a
Nucleolus
GFP::CEC-5
mCherry::FIB-1
Merge
Oocyte
Soma
5 μm
b
n = 55
n = 52
n = 60
n = 64
nucleolar localization percentage %
tissue:
oocyte
embryo
soma
late embryo
GFP::CEC-5
GFP::CEC-8
c
18S
5.8S
26S
18S
CEC-5
Chromodomain proteins
input
CEC-10
CEC-2
CEC-7
CEC-8
CHD-1
CHD-3
CHD-7
LET-418
SET-31
input 1
input 2
input 3
input 4
input 5
-0.5kb
0.5kb
d
met-2 / WT
cec-5(ust240) / WT
cec-5(ust283) / WT
Relative amount of RNA
eft-3
#1
#2
#3
5.8S rRNA
18S rRNA
26S rRNA
e
met-2 / WT
cec-5(ust240) / WT
cec-5(ust283) / WT
Relative amount of RNA
eft-3
#1
#2
#3
#4
pre-rRNA
f
WT
met-2
cec-5(ust240)
cec-5(ust283)
mCherry::Fib-1
10 μm
Oocyte
Embryo
Soma

Supplementary Figure 20. CEC-5 binds rDNA and accumulates in nucleoli. (a) CEC-5 localized to the nucleolus in oocytes (upper panel) and somatic cells (lower panel) (scale bar, 5 μm). The nucleolus is marked by mCherry::FIB-1. **(b)** Quantification of the nucleolar localization of CEC-5 and CEC-8 in the indicated cells. n, number of animals scored. **(c)** ChIP-seq signals of the indicated chromodomain proteins on 18S and 26S rDNA. **(d, e)** Relative abundance of rRNA and pre-rRNA quantified by real-time PCR in indicated animals. Mean ± s.e.m. One-sided t-test was performed. $^{**}p < 0.01$, $^{*}p < 0.05$. *met-2*, pre-rRNA#3, p=0.0180; *cec-5(ust240)*, pre-rRNA#2, p=0.0415; pre-rRNA#4, p=0.0321; *cec-5(ust283)*, pre-rRNA#1, p=0.0102; pre-rRNA#2, p=0.0329; pre-rRNA#4, p=0.00928. n = 3. **(f)** Representative images showing nucleolar morphology in indicated animals. Oocyte (upper panel); Embryo (middle panel); Soma (lower panel). Nucleolus is shown by mCherry::FIB-1 (scale bar, 10 μm). Source data are provided as a Source Data file.

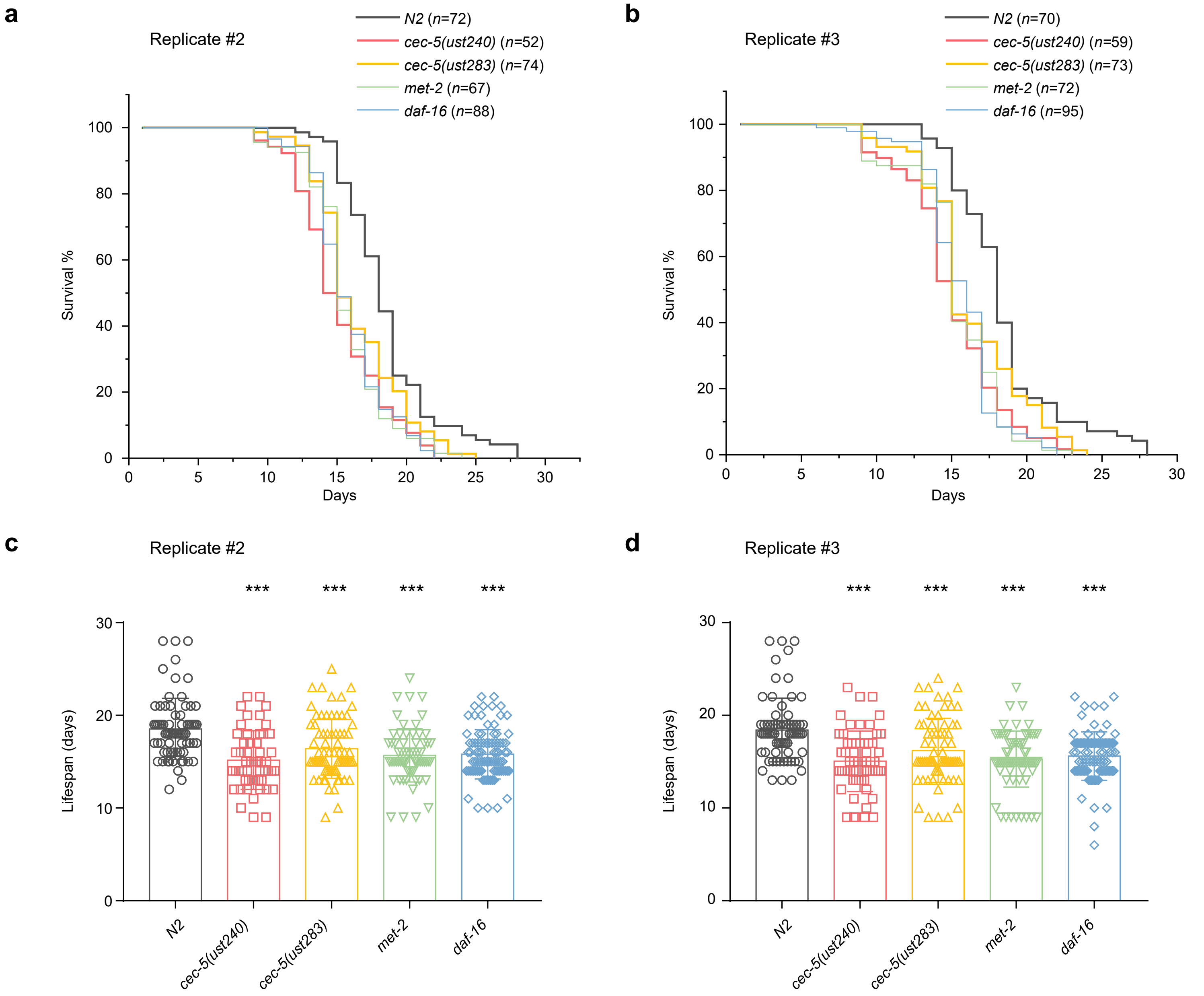

Supplementary Figure 21. CEC-5 is required for lifespan. (a, b) Two replicates of survival curves of the indicated animals. n, the number of animals tested. **(c, d)** Histogram displaying lifespan of the indicated animals. Mean ± s.e.m. of animals. The number of animals tested of each strain is indicated in **Supplementary Figs. 21a-b**. Asterisks indicate significant differences using one-sided t-tests. ***$p < 0.001$. **(c)** *cec-5(ust240)*, $p=4.37 \times 10^{-8}$; *cec-5(ust283)*, $p= 5.71 \times 10^{-5}$; *met-2*, $p=1.34 \times 10^{-7}$; *daf-16*, $p=1.76 \times 10^{-8}$. **(d)** *cec-5(ust240)*, $p=4.62 \times 10^{-8}$; *cec-5(ust283)*, $p= 0.000106$; *met-2*, $p=2.70 \times 10^{-8}$; *daf-16*, $p=6.75 \times 10^{-9}$. Source data are provided as a Source Data file.

**Supplementary Table 1. Homologs of chromodomain proteins in humans and *C. elegans***		
**Family**	***H. sapiens* homolog**	***C. elegans* homolog**
CHD	CHD1	CHD-1
	Mi-2 (CHD-3/CHD-4)	CHD-3
	Mi-2 (CHD-3/CHD-4)	LET-418
	CHD5	CHD-7
HP1	HP1	HPL-1
	HP1	HPL-2
Msl-3 homologs	MRG15	MRG-1
Histone acetyltransferase	MYST1	MYS-1
	MYST1	MYS-2

Supplementary Table 2. Chromodomain sequence identity of C. elegans chromodomain proteins and human HP1α.		
**chromodomain protein**		**% query coverage,% identity**
CEC-1		84,42
CEC-2 isoform a		/
CEC-3		85,37
CEC-4	Sequence from UniProt	100,37
	Sequence from reference (Gonzalez-Sandoval, A. *et al*. *Cell* 2015)	90, 42
CEC-5	Sequence from UniProt	80,25
	Fragment used in this work	79,25
CEC-6		42,52
CEC-7 isoform a	chromodomain 1	/
	chromodomain 2	/
	chromodomain 3	/
CEC-7 isoform b	chromodomain 1	/
	chromodomain 2	/
	chromodomain 3	/
CEC-8		46,37
CEC-10		61,35
CHD-1	chromodomain 1	28,46
	chromodomain 2	84,32
CHD-3	chromodomain 1	/
	chromodomain 2	26,59
CHD-7	chromodomain 1	36,41
	chromodomain 2	30,52
HERI-1		54,41
LET-418	chromodomain 1	/
	chromodomain 2	38,46
MRG-1 isoform a		/
MRG-1 isoform b		/
MYS-1		/
MYS-2 isoform a		/
MYS-2 isoform b		/
UAD-2 isoform b		71,29
SET-31		94,24

Supplementary Table 3. Chromodomain protein ChIP-seq quality control (FRiP, NSC, and RSC)

ChIP-seq Sample name	FRiP	cc(fragment length)	min(cc)	cc(read length)	NSC	RSC
CHD-1 GFP(A)	0.074895674	0.619339428	0.609412062	0.616129979	1.016290072	1.477744687
CHD-3 GFP(A)	0.102479659	0.676084777	0.660013748	0.669349117	1.024349537	1.721520365
CHD-3 SDQ3907(A)	0.1740017	0.732541926	0.720240121	0.72584227	1.017080144	2.195908008
CHD-7 GFP(A)	0.039469327	0.712840795	0.702964928	0.709623212	1.014048876	1.483245134
LET-418GFP(A)	0.084176318	0.663556132	0.651531276	0.659435209	1.018456299	1.521376239
LET-418(A)	0.2402919	0.854873281	0.838029985	0.855102127	1.020098679	0.986595332
CEC-2 GFP(A)	0.022287441	0.688750244	0.684385938	0.690722024	1.006376965	0.68880148
CEC-5 GFP(A)	0.072147357	0.608335869	0.594056577	0.603954213	1.024036923	1.442697111
CEC-8 GFP(E)	0.086486149	0.740697545	0.73523405	0.742853457	1.007430961	0.717049878
CEC-10 GFP(A)	0.036962366	0.582747857	0.572380503	0.582507675	1.018112696	1.023716609
UAD-2 GFP(A)	0.115339338	0.581590751	0.549985156	0.564657295	1.057466269	2.154123239
HPL-2(L3)	0.245705483	0.891394443	0.845525438	0.875176391	1.054249113	1.546965614
HPL-2(A)	0.343973345	0.638746639	0.598282572	0.620356606	1.067633705	1.833107038
SET-31 GFP(A)	0.022882817	0.760747628	0.752521694	0.758929174	1.010931159	1.283801753
CEC-7 GFP(A)	0.032348088	0.725760449	0.719977354	0.724670878	1.008032329	1.232143484
CEC-7 SDQ5413(A)	0.065048357	0.816710362	0.809347012	0.814153866	1.009097889	1.531844004
CEC-7 SDQ5421(A)	0.236059278	0.796855109	0.787884317	0.793124235	1.011385926	1.712010068
MRG-1(L4)	0.40692924	0.573851749	0.551586463	0.556981538	1.040365904	4.126964993

Supplementary Table 4. Coordinates of chromosome arms and central regions

Chromosome	Left Arm		Central Region		Right Arm	
	Position (Mb)	Genes/Mb	Position (Mb)	Genes/Mb	Position (Mb)	Genes/Mb
I	0-3.5	140	3.5-11	216.8	11.0-15.0	183.8
II	0.4-4.3	261.8	4.3-12	233.1	12-14.2	186.4
III	0-3.8	164.2	3.8-10.9	225.6	10.9-13.2	141.7
IV	0.8-4.3	162	4.3-10.7	222.3	10.7-17.1	166.4
V	0.2-5.1	246.3	5.1-15.5	247	15.5-20.9	219.6
X	0-2.0	160	2.0-17.8	155.7		

Supplementary Table 5. Percentage of shared ChIP-seq peaks of chromodomain proteins and histone modifications

1. Percentage of chromodomain protein peaks covered by histone modifications on chromosome arms

Percentage	H3K9me2	H3K9me3	H3K27me3	H3K4me3	H3K36me3	H3K79me3
CHD-1 GFP(A)	0.116359447	0.107142857	0.134792627	**0.501152074**	0.179723502	**0.351382488**
CHD-3 GFP(A)	0.107191316	0.078697422	0.073270014	**0.499321574**	0.066485753	0.251017639
CHD-3 SDQ3907(A)	0.142284569	0.077154309	0.038076152	**0.381763527**	0.04509018	0.245490982
CHD-7 GFP(A)	0.187435633	0.171987642	**0.306900103**	0.284243048	0.151390319	**0.354273944**
LET-418GFP(A)	0.16468254	0.10515873	0.128968254	**0.473214286**	0.109126984	**0.308531746**
LET-418(A)	**0.408372093**	0.046511628	0.054573643	0.199379845	0.074728682	0.120620155
CEC-2 GFP(A)	0.08411215	0.08411215	0.112149533	0.261682243	0.046728972	0.177570093
CEC-5 GFP(A)	**0.443389335**	0.086924763	0.149013879	0.266617969	0.119795471	0.26807889
CEC-8 GFP(E)	**0.490478781**	0.0914037	0.146898803	0.085146899	0.088683351	0.221708379
CEC-10 GFP(A)	0.155963303	0.155963303	0.197247706	0.28440367	0.064220183	0.174311927
UAD-2 GFP(A)	0.148303115	0.121338912	**0.333333333**	0.151092515	0.087866109	0.162715016
HPL-2(L3)	**0.44586561**	0.094757701	0.119257791	0.164294722	0.082147361	0.232210413
HPL-2(A)	**0.337876844**	0.059653624	0.033354715	0.166613214	0.11898653	0.147690827
SET-31 GFP(A)	0.208029197	0.187956204	**0.357664234**	0.277372263	0.149635036	**0.357664234**
CEC-7 GFP(A)	0.089337176	0.083573487	0.129682997	**0.596541787**	0.123919308	0.288184438
CEC-7 SDQ5413(A)	0.122326454	0.009380863	0.007879925	0.190619137	**0.492307692**	**0.541838649**
CEC-7 SDQ5421(A)	0.189347337	0.009152288	0.006751688	0.200150038	**0.388897224**	**0.401350338**
MRG-1(L4)	0.03603182	0.021057557	0.007019186	**0.571829668**	**0.469817501**	**0.708937763**

Annotation: Percentage ⩾0.300000 is shown in **bold**

2. Percentage of chromodomain protein peaks covered by histone modifications on chromosome centers

Percentage	H3K9me2	H3K9me3	H3K27me3	H3K4me3	H3K36me3	H3K79me3
CHD-1 GFP(A)	0.116062176	0.118134715	0.067357513	**0.421761658**	0.099481865	0.239378238
CHD-3 GFP(A)	0.111935266	0.104517869	0.054619016	**0.314227916**	0.024275118	0.141604855
CHD-3 SDQ3907(A)	0.084577114	0.094527363	0.014262023	0.27893864	0.013598673	0.124709784
CHD-7 GFP(A)	0.121842496	0.109955423	0.203566122	**0.359583952**	0.086181278	0.237741456
LET-418GFP(A)	0.133126935	0.118421053	0.070433437	**0.356037152**	0.044891641	0.188080495
LET-418(A)	0.121709271	0.102632583	0.016787486	**0.30904235**	0.00610454	0.125524609
CEC-2 GFP(A)	0.107526882	0.14516129	0.053763441	**0.317204301**	0.016129032	0.155913978
CEC-5 GFP(A)	0.177554439	0.117252931	0.088777219	**0.413735343**	0.055276382	0.211055276
CEC-8 GFP(E)	**0.670588235**	0.141176471	0.035294118	0.105882353	0.011764706	0.223529412
CEC-10 GFP(A)	0.210884354	0.108843537	0.149659864	**0.408163265**	0.06122449	0.176870748
UAD-2 GFP(A)	0.211314476	0.126455907	**0.381031614**	0.194675541	0.053244592	0.144758735
HPL-2(L3)	0.198695652	0.122608696	0.059130435	0.249130435	0.033478261	0.209130435
HPL-2(A)	0.211455847	0.159427208	0.023866348	0.274940334	0.035322196	0.183770883
SET-31 GFP(A)	0.237179487	0.147435897	**0.307692308**	**0.394230769**	0.115384615	0.266025641
CEC-7 GFP(A)	0.17765043	0.143266476	0.100286533	**0.47277937**	0.080229226	0.209169054
CEC-7 SDQ5413(A)	0.100083403	0.097164304	0.007089241	0.141993328	**0.446413678**	**0.360091743**
CEC-7 SDQ5421(A)	0.122872661	0.105853953	0.007483404	0.218467109	**0.349668075**	**0.337477369**
MRG-1(L4)	0.101155439	0.095923261	0.008938304	0.296054066	**0.409635928**	**0.435796817**

Annotation: Percentage ⩾0.300000 is shown in **bold**

3. Percentage of histone modification peaks covered by chromodomain proteins on chromosome arms

Percentage	H3K9me2	H3K9me3	H3K27me3	H3K4me3	H3K36me3	H3K79me3
CHD-1 GFP(A)	0.116133289	0.081771721	0.070423981	0.251396648	0.152002995	0.162042948
CHD-3 GFP(A)	0.104915869	0.065076661	0.057180988	0.26222067	0.141145638	0.157515961
CHD-3 SDQ3907(A)	0.115968327	0.067120954	0.046812442	0.274441341	0.135342568	0.153221126
CHD-7 GFP(A)	0.133949192	0.130494037	0.108305102	0.17527933	0.134593785	0.131979106
LET-418GFP(A)	0.122731772	0.073253833	0.067446874	0.269553073	0.142081617	0.155890888
LET-418(A)	0.22517321	0.046678024	0.065599014	0.224162011	0.144515163	0.127568195
CEC-2 GFP(A)	0.115143517	0.097103918	0.102350888	0.127793296	0.112317484	0.109228091
CEC-5 GFP(A)	0.147641043	0.089608177	0.078944667	0.198673184	0.164170723	0.158676727
CEC-8 GFP(E)	0.250412405	0.119931857	0.10122164	0.100209497	0.181767128	0.183168891
CEC-10 GFP(A)	0.114978555	0.110391823	0.107073196	0.119064246	0.118869337	0.108995937
UAD-2 GFP(A)	0.131639723	0.13560477	0.159839852	0.151885475	0.102583302	0.090307603
HPL-2(L3)	0.280600462	0.126746167	0.101940253	0.192039106	0.141707226	0.194428323
HPL-2(A)	0.259155394	0.073935264	0.050508161	0.168994413	0.151067016	0.148229832
SET-31 GFP(A)	0.12817552	0.121635434	0.111076891	0.143156425	0.130475477	0.126523506
CEC-7 GFP(A)	0.111514352	0.090971039	0.08048455	0.206354749	0.154998128	0.160882182
CEC-7 SDQ5413(A)	0.113988783	0.045655877	0.027820552	0.173184358	0.281916885	**0.305745792**
CEC-7 SDQ5421(A)	0.156713956	0.048040886	0.028128529	0.192388268	**0.354548858**	**0.431804991**
MRG-1(L4)	0.071758496	0.026916525	0.021045067	**0.394902235**	0.258517409	**0.313058619**

Annotation: Percentage ⩾0.300000 is shown in **bold**

4. Percentage of histone modification peaks covered by chromodomain proteins on chromosome centers

Percentage	H3K9me2	H3K9me3	H3K27me3	H3K4me3	H3K36me3	H3K79me3
CHD-1 GFP(A)	0.110619469	0.189655172	0.044424934	0.201546206	0.157082749	0.163905387
CHD-3 GFP(A)	0.130530973	0.195402299	0.030553117	0.223480762	0.128831897	0.146816714
CHD-3 SDQ3907(A)	0.086283186	0.172413793	0.012467076	0.262495505	0.104387898	0.148379702
CHD-7 GFP(A)	0.126106195	0.137931034	0.071641791	0.152822726	0.130635143	0.136292591
LET-418GFP(A)	0.172566372	0.224137931	0.036874451	0.218806185	0.138248848	0.149838491
LET-418(A)	0.254424779	0.166666667	0.014398595	0.27436174	0.106391505	0.154944253
CEC-2 GFP(A)	0.112831858	0.149425287	0.056716418	0.133225458	0.118813865	0.127435657
CEC-5 GFP(A)	0.192477876	0.201149425	0.043546971	0.170262496	0.1420557	0.150255288
CEC-8 GFP(E)	0.258849558	0.103448276	0.066549605	0.111650485	0.110398718	0.122329895
CEC-10 GFP(A)	0.137168142	0.114942529	0.069183494	0.115426106	0.11420557	0.119516516
UAD-2 GFP(A)	0.258849558	0.252873563	0.110096576	0.120100683	0.104387898	0.11618214
HPL-2(L3)	**0.460176991**	0.281609195	0.038630378	0.214850773	0.124624324	0.155673648
HPL-2(A)	**0.407079646**	**0.327586207**	0.020895522	0.200827041	0.158084552	0.203501094
SET-31 GFP(A)	0.201327434	0.229885057	0.092186128	0.126932758	0.125626127	0.133479212
CEC-7 GFP(A)	0.14159292	0.189655172	0.06356453	0.14095649	0.122420357	0.133687611
CEC-7 SDQ5413(A)	0.054347826	0	0.006267955	0.202237333	0.28970374	**0.338721927**
CEC-7 SDQ5421(A)	0.044247788	0	0.024582968	0.167925207	0.279102384	**0.341044076**
MRG-1(L4)	0.006637168	0.005747126	0.002282704	0.248291981	**0.31797235**	**0.320621027**

Annotation: Percentage ⩾0.300000 is shown in **bold**

Supplementary Table 6. Summary of “Graded histone modification occupied” for chromodomain proteins

Chromosome arms	H3K9me2			H3K9me3			H3K27me3			H3K4me3			H3K36me3			H3K79me3		
	Significant overlap>=0.3	Heatmap detectable	Pearson correlation coefficient r>=0.3	Significant overlap>=0.3	Heatmap detectable	Pearson correlation coefficient r>=0.3	Significant overlap>=0.3	Heatmap detectable	Pearson correlation coefficient r>=0.3	Significant overlap>=0.3	Heatmap detectable	Pearson correlation coefficient r>=0.3	Significant overlap>=0.3	Heatmap detectable	Pearson correlation coefficient r>=0.3	Significant overlap>=0.3	Heatmap detectable	Pearson correlation coefficient r>=0.3
CHD-1 GFP(A)										YES	YES					YES		
CHD-3 GFP(A)										YES	YES	YES						
CHD-3 SDQ3907(A)										YES	YES	YES						
CHD-7 GFP(A)		YES			YES		YES	YES			YES			YES		YES	YES	
LET-418GFP(A)										YES	YES					YES		
LET-418(A)	YES	YES	YES								YES	YES						
CEC-2 GFP(A)											YES							
CEC-5 GFP(A)	YES	YES	YES								YES							
CEC-8 GFP(E)	YES	YES	YES															YES
CEC-10 GFP(A)											YES							
UAD-2 GFP(A)		YES			YES		YES	YES	YES		YES			YES			YES	
HPL-2(L3)	YES	YES	YES															
HPL-2(A)	YES	YES	YES															
SET-31 GFP(A)		YES			YES		YES	YES			YES			YES		YES	YES	
CEC-7 GFP(A)										YES	YES							
CEC-7 SDQ5413(A)		YES											YES	YES	YES	YES	YES	YES
CEC-7 SDQ5421(A)		YES											YES	YES	YES	YES	YES	YES
MRG-1(L4)										YES	YES	YES	YES	YES	YES	YES	YES	YES

Chromosome centers	H3K9me2			H3K9me3			H3K27me3			H3K4me3			H3K36me3			H3K79me3		
	Significant overlap>=0.3	Heatmap detectable	Pearson correlation coefficient r>=0.3	Significant overlap>=0.3	Heatmap detectable	Pearson correlation coefficient r>=0.3	Significant overlap>=0.3	Heatmap detectable	Pearson correlation coefficient r>=0.3	Significant overlap>=0.3	Heatmap detectable	Pearson correlation coefficient r>=0.3	Significant overlap>=0.3	Heatmap detectable	Pearson correlation coefficient r>=0.3	Significant overlap>=0.3	Heatmap detectable	Pearson correlation coefficient r>=0.3
CHD-1 GFP(A)										YES	YES							
CHD-3 GFP(A)										YES	YES	YES						
CHD-3 SDQ3907(A)											YES	YES						
CHD-7 GFP(A)								YES		YES	YES			YES			YES	
LET-418GFP(A)										YES	YES							
LET-418(A)										YES	YES	YES						
CEC-2 GFP(A)										YES	YES							
CEC-5 GFP(A)										YES	YES							
CEC-8 GFP(E)	YES	YES	YES															
CEC-10 GFP(A)										YES	YES							
UAD-2 GFP(A)							YES	YES			YES			YES			YES	
HPL-2(L3)											YES							
HPL-2(A)			YES								YES	YES						
SET-31 GFP(A)							YES	YES		YES	YES			YES			YES	
CEC-7 GFP(A)										YES	YES							
CEC-7 SDQ5413(A)													YES	YES	YES	YES	YES	YES
CEC-7 SDQ5421(A)												YES	YES	YES	YES	YES	YES	YES
MRG-1(L4)												YES	YES	YES	YES	YES	YES	YES

Supplementary Table 7. Summary of subcellular localization of chromodomain proteins					
**Prominent subcelluar localiztion is assigned to one or more of the 3 patterns: 1.Nucleoplasm; 2.Nucleolus; 3.Chromosomes and nuclear puncta**					
	Mitotic cells	Meiotic cells	Oocytes	Early embryos	Late embryos
GFP::CHD-1	Nucleoplasm	Nucleoplasm		Nucleoplasm	Nucleoplasm
GFP::CHD-3			Nucleoplasm	Nucleoplasm	Nucleoplasm
GFP::CHD-7			Nucleoplasm	Nucleoplasm	Nucleoplasm
GFP::LET-418	Nucleoplasm	Nucleoplasm	Nucleoplasm	Nucleoplasm	Nucleoplasm
GFP::CEC-5	Chromosomes and nuclear puncta	Chromosomes and nuclear puncta	Chromosomes and nuclear puncta /Nucleolus	Chromosomes and nuclear puncta	Chromosomes and nuclear puncta /Nucleolus
GFP::CEC-7	Nucleoplasm	Nucleoplasm	Nucleoplasm	Nucleoplasm	
GFP::CEC-8					Nucleoplasm /Nucleolus
GFP::HERI-1	Nucleoplasm	Nucleoplasm			
GFP::CEC-10	Nucleoplasm	Nucleoplasm		Nucleoplasm	Nucleoplasm
GFP::UAD-2	Chromosomes and nuclear puncta	Chromosomes and nuclear puncta		Chromosomes and nuclear puncta	Chromosomes and nuclear puncta
GFP::SET-31	Chromosomes and nuclear puncta	Chromosomes and nuclear puncta			
GFP::CEC-2	Not shown				

Supplementary Table 8. Strains used in this work	
**Strain**	**Genotype**
N2	
SHG1571	ustIS194[gfp::3xflag::chd-1] I
SHG1532	ustIS187[gfp::3xflag::chd-3] X
SHG1572	ustIS195[gfp::3xflag::chd-7] I
SHG1674	ustIS210[gfp::3xflag::let-418] V
SHG1691	ustIS196[gfp::3xflag::cec-2] IV
SHG1573	ustIS197[gfp::3xflag::cec-5] IV
SHG1396	ustIS158[gfp::3xflag::cec-7] III
SHG1574	ustIS198[gfp::3xflag::cec-8] III
SHG1601	ustIS201[gfp::3xflag::cec-10] I
SHG1533	ustIS188[gfp::3xflag::heri-1] II
SHG1600	ustIS199[gfp::3xflag::set-31] V
SHG1093	ustIS151[gfp::3xflag::uad-2] I
SHG1598	ustIS194[gfp::3xflag::chd-1] I; ItIs37[pie-1p::mCherry::H2B::pie-1 3'UTR + unc-119(+)] IV
SHG1576	ustIS187[gfp::3xflag::chd-3] X; ItIs37[pie-1p::mCherry::H2B::pie-1 3'UTR + unc-119(+)] IV
SHG1599	ustIS195[gfp::3xflag::chd-7] I; ItIs37[pie-1p::mCherry::H2B::pie-1 3'UTR + unc-119(+)] IV
SHG1672	ustIS210[gfp::3xflag::let-418] V; ItIs37[pie-1p::mCherry::H2B::pie-1 3'UTR + unc-119(+)] IV
SHG1648	ustIS197[gfp::3xflag::cec-5] IV; ustIS200[mCherry::H2B] I
SHG1575	ustIS158[gfp::3xflag::cec-7] III; ItIs37[pie-1p::mCherry::H2B::pie-1 3'UTR + unc-119(+)] IV
SHG1650	ustIS198[gfp::3xflag::cec-8] III; ItIs37[pie-1p::mCherry::H2B::pie-1 3'UTR + unc-119(+)] IV
SHG1653	ustIS201[gfp::3xflag::cec-10] I; ItIs37[pie-1p::mCherry::H2B::pie-1 3'UTR + unc-119(+)] IV
SHG1577	ustIS188[gfp::3xflag::heri-1] II; ItIs37[pie-1p::mCherry::H2B::pie-1 3'UTR + unc-119(+)] IV
SHG1671	ustIS199[gfp::3xflag::set-31] V; ItIs37[pie-1p::mCherry::H2B::pie-1 3'UTR + unc-119(+)] IV
SHG1353	ustIS151[gfp::3xflag::uad-2] I; ItIs37[pie-1p::mCherry::H2B::pie-1 3'UTR + unc-119(+)] IV
SHG1602	ustIS197[gfp::3xflag::cec-5] IV; ustIS036[mCherry::FIB-1] II
SHG1652	ustIS198[gfp::3xflag::cec-8] III; ustIS075[mCherry::SUSI-2] II
SHG1695	ustIS197[gfp::3xflag::cec-5] IV; met-2(n4256) III; set-25(n5021) III
SHG1755	ustIS197[gfp::3xflag::cec-5] IV; met-2(n4256) III
SHG1774	ustIS197[gfp::3xflag::cec-5] IV; set-25(n5021) III
SHG1603	cec-5(ust240) IV
SHG1763	ustIS197[gfp::3xflag::cec-5(ust283)] IV
CB1370	daf-2(e1370) III
CF1038	daf-16(mu86) I
MT13293	met-2(n4256) III
SHG1607	cec-8(ust234) III
SHG1668	cec-10(ust273) I
RB2613	chd-1(ok2798) I
HX103	chd-3(eh4) X
VC606	chd-7(gk290) I
VC978	set-31(ok1482) V

Supplementary Table 9. Primers for PCR amplification of chromodomain gene sequences	
**Primer name**	**Primer sequence (5' to 3')**
cec2 left F	gggtaacgccagCACGTGtgaaaggtctcggaaggaaaat
cec2 left R	tcatggtctttgtagtccatTGTCGATTGCGACATtggtg
cec2 right F	AAGGAGGTGGAGGTGGAGCTATGTCGCAATCGACATCATC
cec2 right R	cagcggataacaatttcacaGGCGCCCAAATTCTCCAAATT
cec5 left F	gggtaacgccagCACGTGtgaattcttacccgatatggctat
cec5 left R	tcatggtctttgtagtccatTGATTTCTTTGCCATttcgaaa
cec5 right F	AAGGAGGTGGAGGTGGAGCTATGGCAAAGAAATCATCCGG
cec5 right R	cagcggataacaatttcacataaatgagcagaacgaaaagaatt
cec7 left F	gggtaacgccagCACGTGtgtggaaaatgacgaaggcgat
cec7 left R	tcatggtctttgtagtccatACGATTAGGAGCCATctgta
cec7 right F	AAGGAGGTGGAGGTGGAGCTATGGCTCCTAATCGTACTCC
cec7 right R	cagcggataacaatttcacaCTCACCGATTATGAATTGTTCT
cec8 left F	gggtaacgccagCACGTGtgccatatcccaccactaacc
cec8 left R	tcatggtctttgtagtccatGGATTTTCTGGCCATggttg
cec8 right F	AAGGAGGTGGAGGTGGAGCTATGGCCAGAAAATCCAAAGGAA
cec8 right R	cagcggataacaatttcacaTTCATTGTCAGAGGACTCTTCT
cec10 left F	gggtaacgccagCACGTGtggtgccgttgacatctggt
cec10 left R	tcatggtctttgtagtccatGCTGGAGCTTGACATtctgaaa
cec10 right F	AAGGAGGTGGAGGTGGAGCTATGTCAAGCTCCAGCGAGA
cec10 right R	cagcggataacaatttcacatgaaagttcacgggtttttgag
chd1 left F	gggtaacgccagCACGTGtggctttctgaaaggtttttcaaa
chd1 left R	tcatggtctttgtagtccatAGACTGATTCCACATatctgaa
chd1 right F	AAGGAGGTGGAGGTGGAGCTATGTGGAATCAGTCTGAATCAT
chd1 right R	cagcggataacaatttcacaCCATCAGCAGCTCGATCTCT
chd3 left F	gggtaacgccagCACGTGtgtttgtttgcatgttgctatttt
chd3 left R	tcatggtctttgtagtccatTTGATCGTCTGACATtgttctt
chd3 right F	AAGGAGGTGGAGGTGGAGCTATGTCAGACGATCAACACGAAT
chd3 right R	cagcggataacaatttcacaTTCCGCCAGTACATGCGA
chd7 left F	gggtaacgccagCACGTGtgatcgagacgcagttaatcac
chd7 left R	tcatggtctttgtagtccatTTCATCCATtttgtgtgtttggt
chd7 right F	AAGGAGGTGGAGGTGGAGCTATGGATGAAGgtgagtctttttg
chd7 right R	cagcggataacaatttcacaTATCCGGGTGGCCCTTGG
heri1 left F	gggtaacgccagCACGTGtgaactagtgtttccctttctgtt
heri1 left R	tcatggtctttgtagtccatCGAATCTTGCGTCATtgtaagt
heri1 right F	AAGGAGGTGGAGGTGGAGCTATGACGCAAGATTCGGATTCTG
heri1 right R	cagcggataacaatttcacaGTGAAGTCTAGCTCTCAATAGC
let418 left F	gggtaacgccagCACGTGtggggcgaatctaaagcgtt
let418 left R	tcatggtctttgtagtccatTTCTTCAGTTGACATtgttgaat
let418 right F	AAGGAGGTGGAGGTGGAGCTATGTCAACTGAAGAAGATCCGT
let418 right R	cagcggataacaatttcacaAGCATCGGAATCATTTTTGCG

Supplementary Table 10. sgRNA targeted sequences for CRISPR/Cas9-directed gene editing technology

Sg name	Targeted sequence (5' to 3')
gfp-chd-1-sg#1	gtctttttggaatttttacg
gfp-chd-1-sg#2	TCACTGGTACTGTTTCCTTC
gfp-chd-1-sg#3	TTTTTCATCATTATCTTCAC
gfp-chd-3-sg#1	ccaaatttccatcttgagaa
gfp-chd-3-sg#2	CGGTGATGAGACAATGGAGG
gfp-chd-3-sg#3	TGGTTTTTTCTTCTCAGAAG
gfp-chd-7-sg#1	gagtactgtaacttcaatat
gfp-chd-7-sg#2	gctgacgcattgtgaagaat
gfp-chd-7-sg#3	aattcactttagaggtgcaa
gfp-let-418-sg#1	agacataaataattgcaaga
gfp-let-418-sg#2	GAAGAAGTTTGTGCTGCTAT
gfp-let-418-sg#3	TCAAGGAATTTCTAATCTGA
gfp-cec-2-sg#1	gagaggattaaccaaggagt
gfp-cec-2-sg#2	TCACGAGAAATTGCAACTGA
gfp-cec-2-sg#3	AGTTGCAATTTCTCGTGAAG
gfp-cec-5-sg#1	gaaagagagtatacgtatga
gfp-cec-5-sg#2	atttatgtatagtcattgag
gfp-cec-5-sg#3	GTGCACAACCTGGAAGTGAG
gfp-cec-7-sg#1	ggaagttcttctccaaaata
gfp-cec-7-sg#2	CCCTACGACGCCACAATTAT
gfp-cec-7-sg#3	TGTTACTGCGTTCACTTCAA
gfp-cec-8-sg#1	gacaaaggaaggagtaacgc
gfp-cec-8-sg#2	accATGGCCAGAAAATCCAA
gfp-cec-8-sg#3	TTTGGAGTTTCACATCAACT
gfp-heri-1-sg#1	tgaaagattaaggcaaatta
gfp-heri-1-sg#2	acttacaATGACGCAAGATT
gfp-heri-1-sg#3	aattaaagccgtacCTGACA
gfp-cec-10-sg#1	aattagaaaggatattacaa
gfp-cec-10-sg#2	AGTCAACGTAGACACTGAAG
gfp-cec-10-sg#3	AAGAAATAGACGAGGATCAC
gfp-set-31-sg#1	gatgatttctgcaaaggatg
gfp-set-31-sg#2	aatttagatgatttctgcaa
gfp-set-31-sg#3	AAAAGAATGCATCCAAGTCA
GFP-F	atggactacaaagaccatgacgg
GFP-R	AGCTCCACCTCCACCTCCTTTG
vector-F	tgtgaaattgttatccgctgg
vector-R	caCACGTGctggcgttacc
del-cec-5-sg#1	GTGCACAACCTGGAAGTGAG
del-cec-5-sg#2	TATAATGTCCTCCTTTCAAA
del-cec-5-sg#3	CAAGACAATACTAGTGGAAG
del-cec-5-sg#4	aatgtggaacgttacagaat
del-cec-8-sg#1	gacaaaggaaggagtaacgc
del-cec-8-sg#2	TTTGGAGTTTCACATCAACT
del-cec-8-sg#3	tcgatagtttaaagttcaaa
del-cec-8-sg#4	GAGCAAGCCGAGGAGACCAA
del-cec-10-sg#1	aattagaaaggatattacaa
del-cec-10-sg#2	attagaaaggatattacaac
del-cec-10-sg#3	AGTCAACGTAGACACTGAAG
del-cec-10-sg#4	AAGAAATAGACGAGGATCAC
del-cec-10-sg#5	AGAAATAGACGAGGATCACT

Supplementary Table 11. Primers used for quantitative real time PCR

Primer name	Sequence (5' to 3')
eft-3-F	GTAAGGGATCTTTCAAGTACGC
eft-3-R	CATCGATGATGGTGATGTAGTAC
18S#1-F	ATACCTGATTGATTCTGTC
18S#1-R	TTCCGCAGTTATCCATAT
18S#2-F	CATTAGGACTGACAGATTGAA
18S#2-R	TCGCTCGTTATCGGAATA
18S#3-F	AACCGTTGAAATTCTTCCA
18S#3-R	GCTGATGACTCACACTTAC
26S#1-F	TGAACTCAGTCGTGATTACC
26S#1-R	CACTCGCCGTTACTAAGG
26S#2-F	GTTTAGTTACGCTAGTGTG
26S#2-R	CAATCCGTGTTTCAAGAC
26S#3-F	GGAATCCGACTGTCTAAT
26S#3-R	CGCTTACTCGAATTACTAC
5.8S-F	TTGCTGCGTTACTTACCA
5.8S-R	GAACCAGACGTACCAACT
pre-rRNA#1-F	TCATTGCGCCGATCCATAGAT
pre-rRNA#1-R	CAAGACCAATACCGCAACATCA
pre-rRNA#2-F	CACGGTCAGTTGAGTGTCGA
pre-rRNA#2-R	GTAGTTTTGGAATAACTGCCACG
pre-rRNA#3-F	TATCGGAGGAGCTGCCAAATGG
pre-rRNA#3-R	GGAGCGACGACACATTCTGAC
pre-rRNA#4-F	GGACACACCACCAAAGTCTCAA
pre-rRNA#4-R	TTGAGAGACGGCAGACAACG
